# A genome-wide association study of mitochondrial DNA copy number in two population-based cohorts

**DOI:** 10.1186/s40246-018-0190-2

**Published:** 2019-01-31

**Authors:** Anna L. Guyatt, Rebecca R. Brennan, Kimberley Burrows, Philip A. I. Guthrie, Raimondo Ascione, Susan M. Ring, Tom R. Gaunt, Angela Pyle, Heather J. Cordell, Debbie A. Lawlor, Patrick F. Chinnery, Gavin Hudson, Santiago Rodriguez

**Affiliations:** 1 MRC Integrative Epidemiology Unit, University of Bristol, Bristol, UK; 2 Population Health Sciences, Bristol Medical School, University of Bristol, Bristol, UK; 3 Wellcome Centre for Mitochondrial Research, Newcastle University, Newcastle, UK; 4 Bristol Heart Institute, Translational Health Sciences, Bristol Medical School, University of Bristol, Bristol, UK; 5 Institute of Genetic Medicine, Newcastle University, Newcastle, UK; 6 Department of Clinical Neurosciences and MRC Mitochondrial Biology Unit, University of Cambridge, Cambridge, UK

**Keywords:** Mitochondrial DNA, ALSPAC, Genome-wide association study, Complex traits, Genetic epidemiology

## Abstract

**Background:**

Mitochondrial DNA copy number (mtDNA CN) exhibits interindividual and intercellular variation, but few genome-wide association studies (GWAS) of directly assayed mtDNA CN exist.

We undertook a GWAS of qPCR-assayed mtDNA CN in the Avon Longitudinal Study of Parents and Children (ALSPAC) and the UK Blood Service (UKBS) cohort. After validating and harmonising data, 5461 ALSPAC mothers (16–43 years at mtDNA CN assay) and 1338 UKBS females (17–69 years) were included in a meta-analysis. Sensitivity analyses restricted to females with white cell-extracted DNA and adjusted for estimated or assayed cell proportions. Associations were also explored in ALSPAC children and UKBS males.

**Results:**

A neutrophil-associated locus approached genome-wide significance (rs709591 [*MED24*], *β* (change in SD units of mtDNA CN per allele) [SE] − 0.084 [0.016], *p =* 1.54e−07) in the main meta-analysis of adult females. This association was concordant in magnitude and direction in UKBS males and ALSPAC neonates. SNPs in and around *ABHD8* were associated with mtDNA CN in ALSPAC neonates (rs10424198, *β* [SE] 0.262 [0.034], *p =* 1.40e−14), but not other study groups. In a meta-analysis of unrelated individuals (*N* = 11,253), we replicated a published association in *TFAM* (β [SE] 0.046 [0.017], *p =* 0.006), with an effect size much smaller than that observed in the replication analysis of a previous in silico GWAS.

**Conclusions:**

In a hypothesis-generating GWAS, we confirm an association between *TFAM* and mtDNA CN and present putative loci requiring replication in much larger samples. We discuss the limitations of our work, in terms of measurement error and cellular heterogeneity, and highlight the need for larger studies to better understand nuclear genomic control of mtDNA copy number.

**Electronic supplementary material:**

The online version of this article (10.1186/s40246-018-0190-2) contains supplementary material, which is available to authorized users.

## Introduction

Mitochondria are the cellular organelles responsible for producing adenosine triphosphate (ATP), a ubiquitous substrate required for metabolism. ATP is the final product of the series of redox reactions that are facilitated by the complexes of the respiratory chain (RC), located on the cristae, the folded inner membrane of mitochondria.

Mitochondria possess their own genome (mtDNA), an extra-nuclear, double-stranded, circular DNA molecule of ~ 16.6 kb that is inherited maternally. Thirteen subunits contributing to complexes of the RC are encoded by mtDNA, and the entire mitochondrial genome is present at variable copy number in the cell. The relative copy number of mtDNA (mtDNA CN) may reflect differing energy requirements between cells: those from active tissues (e.g. liver, muscle, neuron) are observed to have higher mtDNA CNs compared to endothelial cells, which are comparatively quiescent [1–3].

Several nuclear genes are known to influence the regulation of mtDNA CN, and these are reviewed in detail elsewhere [1, 4–6]. These include *POLG* [4–12] and *POLG2* [4, 5, 12], the catalytic and accessory subunits of DNA polymerase-gamma, the principal enzyme implicated in mtDNA replication. Other regulators include *TFAM* (mitochondrial transcription factor A) [4, 13–17], which initiates mtDNA replication, along with other factors *TFB1M* and *TFB2M* [4, 8, 18]. Regulators of these transcription factors include *PGC-1α* (peroxisome proliferators-activated receptor gamma coactivator 1 alpha) [4, 5, 8] and two nuclear respiratory factors (*NRF-1*, *NRF-2*) [4, 5, 8]. Moreover, maintenance of replication requires an adequate mitochondrial nucleotide supply [19]: nucleotides may be imported from the cytosol or salvaged by specific mitochondrial enzymes. Defective phosphorylation of deoxyribonucleosides by kinases encoded by *DGUOK* (deoxyguanosine kinase) and *TK2* (thymidine kinase) leads to dysfunctional mitochondrial dNTP synthesis and key regulators of dNTP synthesis in the cytosol include the helicase *C10orf2* (alias *TWINK*) [4–10, 12], along with thymidine phosphorylase (*TYMP*) [4, 5, 9] and the target of the p53-transcription factor, p53R2 (encoded by *RRM2B*) [4, 6, 7, 9, 12]. The role of succinyl CoA synthase deficiency as a cause of mtDNA depletion is less well understood, but mutations in the alpha and β subunits of succinyl CoA synthase genes (*SUCLA2*, *SUCGL1*) [6, 7, 9, 10] may be associated with mitochondrial nucleotide depletion [6].

To our knowledge, few genome-wide scans of mtDNA CN have been published, and those that exist are of relatively small sample size [16, 20, 21] or use in silico proxies for mtDNA CN without actual biological measurements [17]. We had access to directly assayed mtDNA CN in a diverse set of study groups and so performed hypothesis-generating genome-wide association studies (GWAS) in ~ 14,000 individual participants from the Avon Longitudinal Study of Parents and Children (ALSPAC) and the UK Blood Service (UKBS) cohort. For our main analyses, the two most comparable study groups of adult females were combined in a joint analysis (*N* = 6799, approximately 10 times larger than previous GWAS of directly assayed mtDNA CN) [20, 21], with results from the other groups presented as opportunistic, secondary analyses. It is known that cellular heterogeneity contributes to mtDNA CN: granulocytes have relatively few mitochondria, whereas lymphocytes are rich in mitochondria, and therefore in mtDNA [22]. Since we also had access to data on white cell proportions, estimated from methylation data in ALSPAC, and assayed directly in UKBS, we performed sensitivity analyses that considered DNA source (whole blood/white cells), and controlled for white cell proportions. Finally, we extracted two SNPs that were robustly related to mtDNA CN in a recent GWAS of mtDNA CN measured in silico [17], and compared our results to those published associations.

## Participants and methods

### Cohort details

ALSPAC is a prospective cohort of mothers and their children. Between 1991 and 1992, 14,541 women living in the former county of Avon, UK, were recruited during pregnancy, of whom 13,761 were enrolled into the study (women were aged between 16 and 43 years at recruitment when samples for mtDNA CN analyses were obtained). Further details are available in the cohort profile papers [23, 24], and the study website contains details of all data that are available through a fully searchable data dictionary: http://www.bristol.ac.uk/alspac/researchers/our-data/. Ethical approval for the study was obtained from the ALSPAC Ethics and Law Committee and the Local Research Ethics Committees.

The UK Blood Service control group is part of the Wellcome Trust Case Control Consortium 2. The UK National Blood Service (UKBS) consists of 3091 unrelated, healthy individuals (aged 17–69 years when samples for mtDNA CN assay were obtained), recruited between September 2005 and February 2006. Informed consent was obtained from all participants in accordance with protocols approved by the Peterborough & Fenland Local Research Ethics Committee in September 2005.

### DNA samples

Blood samples used for mtDNA CN assay were collected from ALSPAC mothers during routine antenatal care. Children included in this study had DNA sampled at either birth (from cord blood, these individuals are hereafter referred to as ALSPAC ‘neonates’) or at a follow-up research clinic assessment at mean age 7 (range 6–9 years, hereafter ALSPAC ‘6–9-year-olds’). Antenatal DNA from mothers was extracted using a phenol-chloroform method [25]. DNA from ALSPAC children was extracted using a phenol-chloroform (ALSPAC neonates) or salting-out method (ALSPAC 6–9-year-olds) [25]. DNA sources used for the mtDNA CN assay varied by age group: DNA from whole blood was used for 6–9-year-olds, ALSPAC mothers’ DNA was extracted from whole blood or white cells, and ALSPAC neonates had DNA extracted from white cells, as described previously [25].

UKBS blood samples were separated by density centrifugation, and white blood cells were retained to perform DNA extractions, as previously described, using a guanidine-chloroform-based method [26, 27]. Thus, in UKBS, the DNA source was white blood cells for all participants. Blood composition information for UKBS samples was provided by Willem Ouwehand at the University of Cambridge as part of an on-going collaboration with Patrick Chinnery. These details are also summarised in Table 1.

**Table 1:** Description of study groups and analysis structure in this paper

Cohort	Group	% female	Source of DNA	Age at DNA assay	Site of assay	Covariates in baseline model	Cell proportions source	Cell proportions included in sensitivity model	*N* (*N* [CC]) main analysis	*N* (*N* [CC]) by subgroups	Meta-analyses (MA) in which each group features
ALSPAC	Mothers^a^(antenatal)	100%	White cells or whole blood [phenol-chloroform extracted]	16–43 years	Bristol	Age, DNA concentration, PC1, PC2, sample type (white cell or whole blood)	Estimated for a subset from Illumina 450 k methylation data	Lymphocytes and neutrophils	5461 (546) (All DNA sources)	2056 (245) [Whole blood samples] 3405 (301) [White cell samples]	MA1 [main] all mothers MA2 [sensitivity] white cell mothers only
	Children^b^	49%	Whole blood [salt-extracted]	6–9 years	Bristol	Age, sex, DNA concentration, PC1, PC2		Lymphocytes, monocytes, and neutrophils	NA	2102 (80)	–
	Neonates^b^	44%	White cells [phenol-chloroform extracted]	0 years	Bristol	Sex, DNA concentration, PC1, PC2		Lymphocytes, monocytes, and granulocytes	NA	3647 (606)	–
UKBS	Female^a^	50%	White cells [guanidine-chloroform extracted]	17–69 years	Newcastle	Age, PC1–PC4	Derived from full blood counts^c^	Lymphocytes and neutrophils	1338 (1138) [Females only]	–	MA1 [main] females only MA2 [sensitivity] females only
	Male								NA	1333 (1122) [Males]	–

*CC* cell composition adjusted model

^a^Group used in the main meta-analyses

^b^Children and neonates in these secondary analyses are unrelated to each other at IBD > 0.125, but some are related to the ALSPAC mothers (see ‘Genotype data’ in the ‘Participants and methods’ section)

^c^See Nalls et al. [27]; Gieger et al. [46]

### Genotype data

#### ALSPAC

ALSPAC mothers were genotyped on the Illumina Human660W-Quad array (Illumina, San Diego, CA, USA) at the Centre Nationale du Génotypage (CNG). ALSPAC children were genotyped with the Illumina HumanHap550-Quad array, by the Wellcome Trust Sanger Institute, Cambridge, UK, and the Laboratory Corporation of America, Burlington, NC, USA, using support from 23andMe. Genotypes were called using Illumina GenomeStudio®. Quality control (QC) was performed using PLINK v1.07 [28], phasing using ShapeIT (v2.r644) [29], and imputation was to the Haplotype Reference Consortium (v1.0), performed using IMPUTE (v3) (http://mathgen.stats.ox.ac.uk/impute/impute.html). The genome build used was GRCh37. Further details of genotype QC are given in Additional file 1.

GWAS were run separately in 5461 ALSPAC mothers, 3647 6–9-year-olds, and 2102 neonates (see Additional file 1 for details of selection into the study). Relatedness within each group of participants (mothers, neonates, and 6–9-year-olds) was assessed by identical-by-descent (IBD) proportions from a genetic relatedness matrix, calculated using the GCTA standard algorithm [30], based on 1.1 million HapMap3 best-guess tag SNPs, present at a combined allele frequency of > 0.01 and imputation quality > 0.8 in 17,842 individuals. Within each group (mothers, 6–9-year-olds, and neonates), participants were unrelated (IBD > 0.125; i.e. first-cousin level). A subset of children was related to the 5461 ALSPAC mothers: there were 1611 mother/6–9-year-old pairs related at IBD > 0.125 (1570 pairs IBD > 0.45) and 869 mother-neonate pairs related at IBD > 0.125 (839 IBD > 0.45). For some sensitivity analyses, a GWAS of a subset of 2833 mothers, who are unrelated to any 6–9-year-olds or neonates at IBD > 0.125, is used. SNPs were filtered by MAF < 0.01 and imputation score < 0.8 in all study groups [31], leaving 7,360,988; 7,410,776; and 7,361,275 SNPs in ALSPAC mothers, 6–9-year-olds, and neonates, respectively.

#### UKBS

The UKBS cohort was genotyped using the Illumina 1.2 M Duo platform. Raw genotype data (called using Illuminus [32], http://www.sanger.ac.uk/science/tools/illuminus) were downloaded from the European Genotype Archive (http://www.ebi.ac.uk/ega). QC was performed using PLINK v1.90 [33], phasing and imputation using EAGLE2 (v2.0.5) [34], and PBWT [35] (imputation was to the Haplotype Reference Consortium (v1.0) performed using the Sanger Imputation Server [https://imputation.sanger.ac.uk/]). The genome build was GRCh37. Further details are given in Additional file 1.

GWAS were run in 2671 UKBS individuals (1333 males, 1338 females). Individuals were unrelated at any level of IBD. After filtering by MAF < 0.01 and imputation quality > 0.8, 7,441,490 variants remained (7,369,986 males only and 7,373,492 females only).

### Assay of mtDNA CN

For mtDNA CN assay details in ALSPAC and UKBS, see Additional file 1. Both cohorts had mtDNA CN assayed by quantitative PCR. Both assays used *B2M* as the single-gene reference, but mtDNA amplicons differed. Raw data are plotted in Additional file 2: Figure S1.

Despite differences in raw mtDNA CNs between study groups, validation analyses of the two adult cohorts suggested relative mtDNA CNs were reliable. Cross-validation of qPCR methodology between centres was performed on 384 random samples (169 from ALSPAC, 185 from UKBS). Samples were assayed by PG and AG in Bristol and RB in Newcastle, using cohort-specific protocols. There was moderate-to-good agreement between *z*-scores of mtDNA CNs assayed in ALSPAC by AG and PG (*r*[Spearman] = 0.68) and between those obtained from the two sets of exchanged plates (ALSPAC *r*[Spearman] = 0.58 [analysts: AG/RB] and UKBS *r*[Spearman] = 0.69 [analysts: PG/RB]) (Additional file 2: Figure S2). However, panel B of Additional file 2: Figure S2 suggests that there may have been some non-linearity when comparing the two assays. To control for absolute differences in mtDNA CNs, *z*-scored phenotypes were used in GWAS (after log-transformation to approximate normality). *Z*-scores were computed separately for ALSPAC mothers, 6–9-year-olds, neonates, and UKBS.

### Statistical analysis

#### Genome-wide association study

GWAS were undertaken separately for ALSPAC mothers, ALSPAC 6–9-year-olds, ALSPAC neonates, UKBS females, and UKBS males. Additive models were fitted, using dosage data for ALSPAC and best-guess data for UKBS. The genome-wide significance threshold was *p =* 5e−08 [36].

##### Main analyses

We had access to several study groups with relevant data. Since these groups were diverse in nature and were of relatively small sample sizes (compared to some complex trait GWAS), we did not consider them as ‘discovery’ and ‘replication’ cohorts. Instead, after validating and harmonising data (see the ‘Assay of mtDNA CN’ section), we considered our main analyses to be hypothesis-generating GWAS of the two most comparable groups, i.e. all adult females (5461 ALSPAC mothers and 1338 UKBS females). This decision also took into account results from some preliminary analyses that suggested some possible differences between UKBS females and males (see Additional file 2: Figures S3 and S4), although we acknowledge that we have insufficient power to detect sex differences that are not potentially due to chance.

Thus, results from ALSPAC mothers and UKBS females were meta-analysed (‘Meta-analysis 1’), using random-effects models in order to protect against possible heterogeneity in effects between cohorts. Since ALSPAC mothers had DNA extracted from two sources, a sensitivity meta-analysis (‘Meta-analysis 2’, *N* = 4743) restricted the ALSPAC mothers to 3405 females with white cell DNA extracted by a phenol-chloroform method (i.e. the most comparable subgroup to UKBS females, all of whom had white cell DNA extracted by a guanidine-chloroform based method [26, 27]). Heterogeneity was assessed using Cochran’s Q statistic [37] and the *I*^2^ statistic [38]. Using the summary statistics from Meta-analysis 1, we estimated the variance explained in mtDNA CN using LD score regression [39].

##### Secondary analyses

Results from GWAS of 3647 ALSPAC children [6–9 years], 2102 ALSPAC neonates, and 1333 UKBS males are presented as secondary analyses. We applied the same *p* value threshold for genome-wide significance and also specifically looked at whether hits identified in these groups showed similar directions and magnitudes of association in the meta-analyses of adult females. However, we did not consider these groups to be suitable replication samples for the main analyses of adult females, given their small sample sizes and sex and age differences.

##### Look-up of top loci from a recent large GWAS of in silico mtDNA CN

A recent large GWAS (using a discovery sample of 10,560 Han Chinese females) identified *TFAM* (mitochondrial transcription factor A) and *CDK6* (Cyclin Dependent Kinase 6), as loci strongly associated with mtDNA CN estimated in silico, from sequence data [17]. The replication sample for that study included 1753 ALSPAC children within the UK10K consortium [40]. There is overlap between that group and the 6–9-year-olds studied in the current analysis [17]. Using all available unrelated participants in the current study (i.e. excluding mother-child duos, *N* = 11,253 total), we meta-analysed the two lead SNPs at these loci and compared our results to those of this previous GWAS [17].

#### Covariates

Covariates included age at mtDNA CN assay, sex, and DNA concentration (ng/μL) [41] (ALSPAC only, measured by a PicoGreen® fluorescence-based method), and principal components (2 in ALSPAC, 4 in UKBS), to adjust for population structure. The ALSPAC mothers’ analysis was adjusted for DNA source (white cells or whole blood) (see Table 1).

For all analyses, effect sizes adjusted for cell proportions are also presented, since cell lineages vary in their average numbers of mitochondria [22]. In ALSPAC, cell proportions have been estimated in the ‘Accessible Resource for Integrated Epigenomics’ (ARIES) subset [42], using DNA methylation data from the Illumina Infinium HumanMethylation450 BeadChip (450 K) array and the Houseman method [43]. Five hundred forty-six mothers, 606 6–9-year-olds, and 82 neonates included in this study had cell proportion data. Proportions were estimated from methylation data derived from the same time points as mtDNA CNs were assayed (antenatally for mothers, at birth and ~ 7 years for children). In this study, we only performed cell proportion-adjusted analyses if participants had mtDNA CN assayed in the same DNA sample type that was used for cellular proportion estimation (i.e. white cells/whole blood) [44]. Lymphocytes (total of CD8T, CD4T, B lymphocytes) and neutrophils (granulocytes for neonates [45]) were included as covariates for mothers, with the addition of monocytes for 6–9-year-olds and neonates (monocytes were not used in the mothers’ analyses since this prevented model convergence). In UKBS, neutrophil and lymphocyte proportions derived from full blood count data were included [27, 46].

#### Software

GWAS were performed with SNPTESTv2.5.0 [47] and meta-analysed with META v1.7.0 [48]. The ‘qqman’ R package [http://cran.r-project.org/web/packages/qqman/] was used to create Manhattan/quantile-quantile (QQ) plots. The ‘bedtools’ ‘clusterBed’ function [https://github.com/arq5x/bedtools2] [49] was used to group SNPs associated at *p* < 1e-06 (except for the 'Meta-Analysis 2', see footnote of Table 2) into 1Mb clusters, which were then annotated with ANNOVAR [50] [http://annovar.openbioinformatics.org/en/latest/]. Regional association plots were produced with LocusZoom v1.3, using an hg19 reference (1000G March 2012) [51].

#### Power

An R implementation of the method used by Genetic Power Calculator [52, 53] was used to determine the minimum detectable effect sizes at 80% power, given the sample size of the study groups used in the main meta-analyses (‘http://www.cureffi.org/2012/12/05/power-for-gwas-and-extreme-phenotype-studies/’). This method requires ‘total QTL variance’ (i.e. the proportion of variance in a quantitative trait locus, *V*_*q*_ explained by the causal variant) as input. Assuming a standardised normal distribution (phenotype in SD units), the following is true:

$$ {V}_q={\beta}^2p $$

where *β* is the effect size (in SD units) and *p* is the minor allele frequency (MAF). Estimated minimal effect sizes for our given sample sizes, a range of *V*_*q*_ values was calculated, with a minimum value of 0.001 (equivalent to an effect size of 0.316 at MAF = 0.01 and 0.045 at MAF = 0.5). Linkage disequilibrium (LD) of 0.8 (measured by the D’ metric) was assumed between causal and tag variants. Power curves are shown in Additional file 2: Figure S5. Heat maps of power by effect size and MAF are shown in Additional file 2 Figure S6. For the largest (*N* = 6799 [5461 + 1338]) and smallest (*N* = 1333) GWAS undertaken in this paper (i.e. the main meta-analysis of adult females and the smallest secondary analysis of UKBS males, respectively), minimum *V*_*q*_ values detectable were 0.0091 and 0.0458. At MAF = 0.25, this is equivalent to effect sizes of 0.191 and 0.428. However, it should be noted that these calculations do not take account of measurement error in mtDNA CN, white cell heterogeneity, covariate adjustment, or the use of random-effects meta-analysis for the main GWAS.

## Results

### Main analysis

**Fig. 1:**
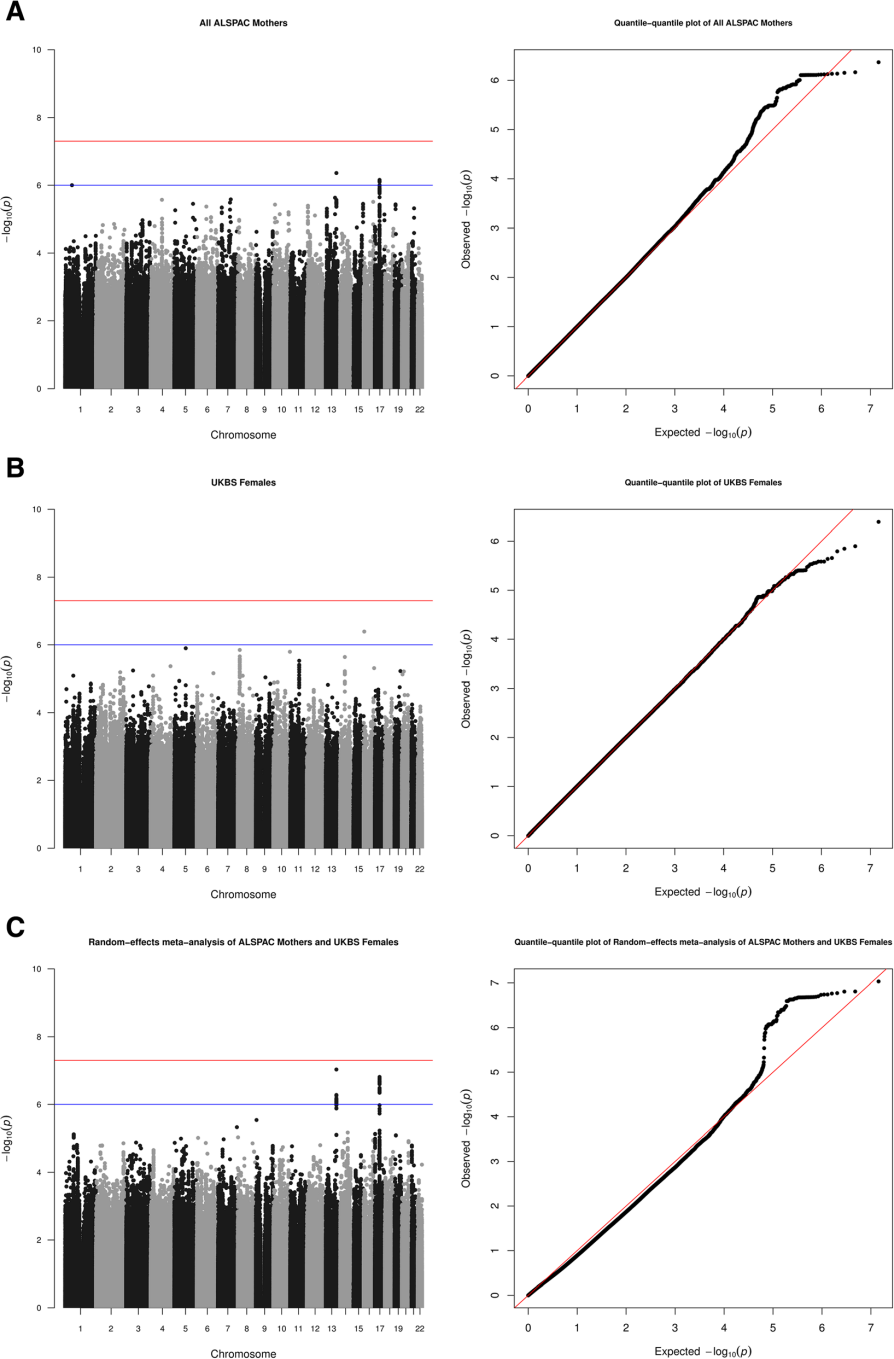
**a** Manhattan (left)/quantile-quantile (QQ) plots (right) for ALSPAC (all mothers). *λ* = 0.995. ‘Minimally adjusted’ refers to the fact that these results are from the analysis that did not adjust for cell proportions. **b** Manhattan (left)/quantile-quantile (QQ) plots (right) for UKBS (females). *λ* = 1.011. ‘Minimally adjusted’ refers to the fact that these results are from the analysis that did not adjust for cell proportions. **c** Manhattan (left)/quantile-quantile (QQ) plots (right) for random-effects meta-analysis of ALSPAC (all mothers) and UKBS (females). *λ* = 0.995 and 1.011 for ALSPAC (all mothers) and UKBS females, respectively, and meta-analyses are corrected for these lambdas. ‘Minimally adjusted’ refers to the fact that these results are from the analysis that did not adjust for cell proportions

**Fig. 2:**
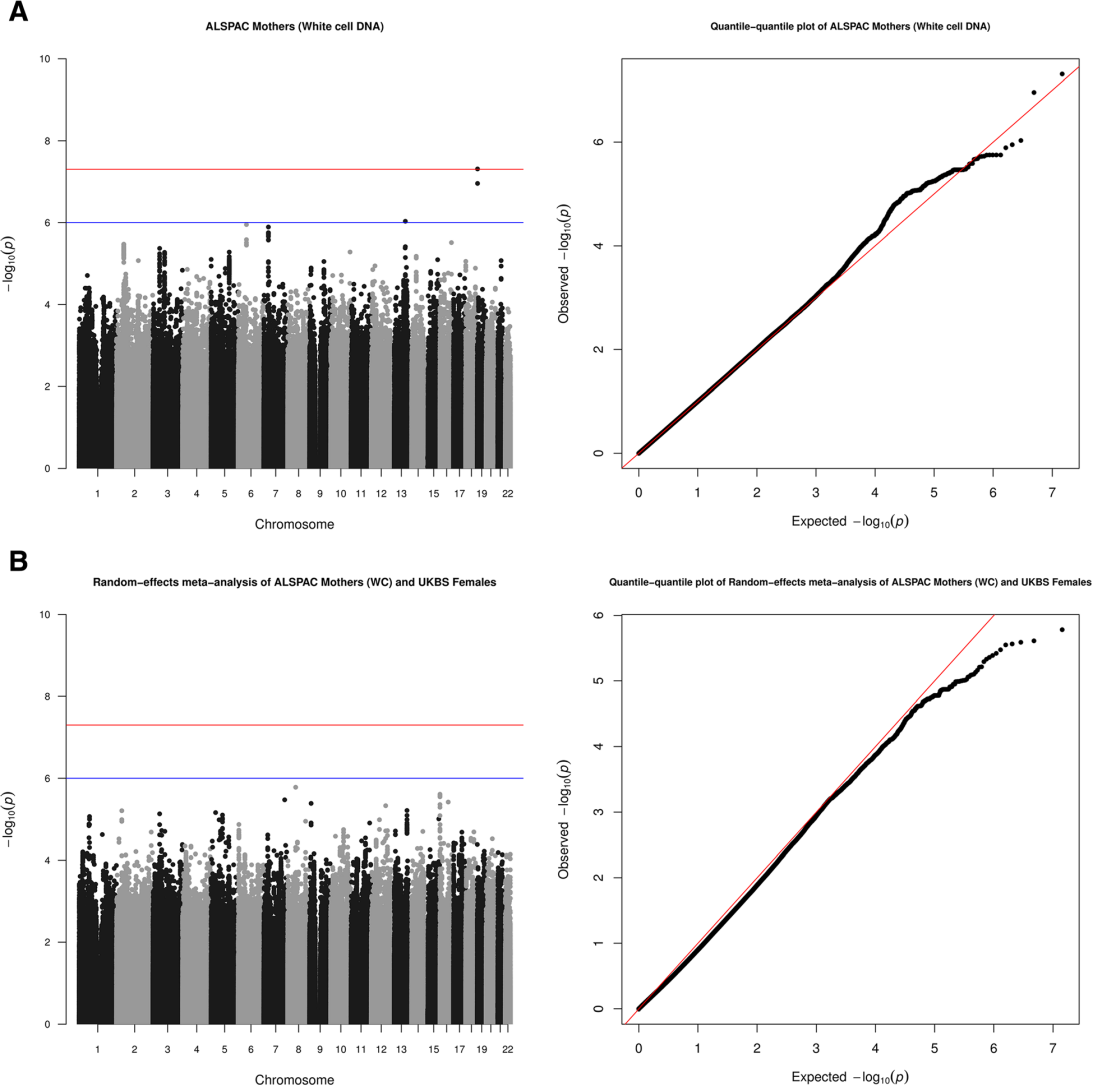
**a** Manhattan (left)/quantile-quantile (QQ) plots (right) for ALSPAC (white cell mothers). *λ* = 0.992. ‘Minimally adjusted’ refers to the fact that these results are from the analysis that did not adjust for cell proportions. **b** Manhattan (left)/quantile-quantile (QQ) plots (right) for random-effects meta-analysis of ALSPAC (white cell mothers) and UKBS females. *λ* = 0.992 and 1.011 for ALSPAC (white cell mothers, see Fig. 2a) and UKBS females (see Fig. 1b), and meta-analyses are corrected for these lambdas. ‘Minimally adjusted’ refers to the fact that these results are from the analysis that did not adjust for cell proportions

**Fig. 3:**
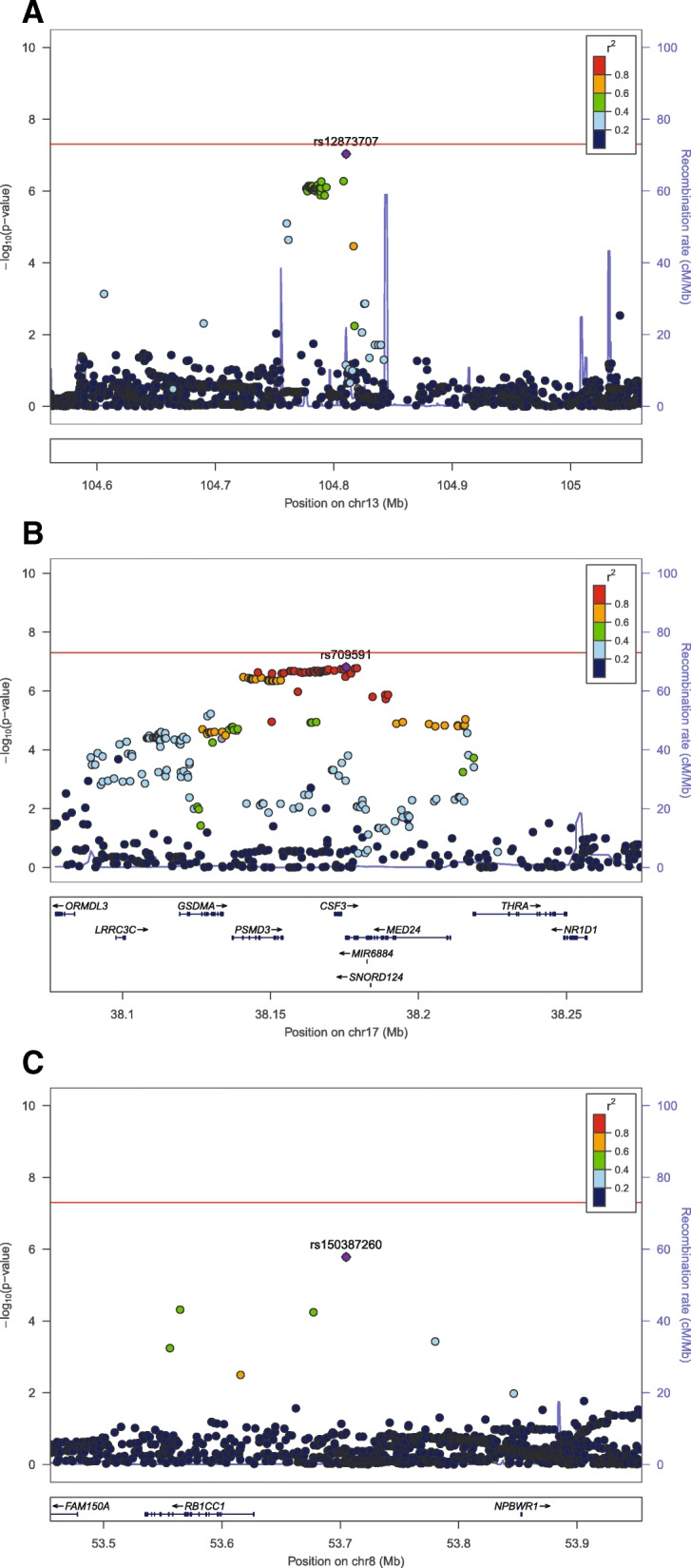
Regional association plots (created with LocusZoom). These three regional association plots (**a**, **b**, and **c**, created with LocusZoom) detail the top loci presented in the meta-analyses of all ALSPAC mothers and UKBS females (**a**, **b**) and of the one locus identified after restriction of the meta-analysis to ALSPAC mothers with DNA extracted from white cells, only (**c**). In each plot, the lead SNP (i.e. the SNP with the lowest *p* value) is annotated in purple, with other SNPs colour coded according to their values of linkage disequilibrium (in *r*^2^) with the lead SNP. Transformed, −log_10_*p* values and recombination rate (in centimorgans per megabase, cM/Mb) are shown on the left and right *y*-axes, respectively. A schematic of the genes in each region, along with coordinates and annotations, is shown at the bottom of each plot, with chromosomal location in megabases (Mb) along the *x*-axis. See Table 2 for more details of each locus

Manhattan/QQ plots for GWAS of ALSPAC mothers (for all 5461 mothers and for 3405 with white cell-extracted DNA), UKBS females, and the two meta-analyses are shown in Figs. 1 and 2. Values of lambda from genomic control calculations are provided in the appropriate figure legend. Regional association plots for loci identified from the meta-analyses are in Fig. 3. For strongest associations from separate GWAS of ALSPAC mothers and UKBS females, see Additional file 3: Tables S1, S2, S3, S4, and S5 and Additional file 1.

Results from the main meta-analysis of adult females (*N* = 6799, ‘Meta-analysis 1’) as well as the analysis restricted to those with white cell-extracted DNA (*N* = 4743, ‘Meta-analysis 2’) are given in Table 2. SNPs in associated regions were clustered into 1 Mb windows [49]. For each analysis, results are shown before and after cell-proportion adjustment. Annotations are given for loci within 200 kb [54]. Coordinates are hg19.

**Table 2:** Summary statistics for top SNPs associated with mtDNA CN in two random-effects meta-analyses

Meta-analysis 1: ALSPAC mothers (*N* = 5461) and UKBS females (*N* = 1338)											
rsID	Cluster	chr:pos_nonEA_EA	*β* (SE) *P*	*Q* *P* (het)	*I* ^2^	*β* (SE) *P* (ALSP)	*β* (SE) *P* (UKBSF)	Location	Lead SNP locus (or distance to genes within 200 kb, in bp)	Genes in this cluster	*β* (SE) *P* (CC)^a^
rs12873707	13:104777684-104810437	13:104810437_T_C	0.159 (0.030) *p =* 9.27E−08	0.169 *p =* 0.681	0	0.165 (0.033) *p =* 4.34E−07	0.131 (0.076) *p =* 0.084	Intergenic	*.*	*.*	0.081 (0.060) *p =* 0.176
rs709591	17:38140926-38179290	17:38175561_T_A	− 0.084 (0.016) *p =* 1.54E−07	0.092 *p* = 0.762	0	− 0.086 (0.018) *p =* 1.05E−06	− 0.073 (0.038) *p =* 0.056	UTR3	*MED24*	*CSF3*, *MED24*, *PSMD3*	− 0.025 (0.033) *p =* 0.453
Meta-analysis 2: ALSPAC mothers (white cell samples only, *N* = 3405) and UKBS females (*N* = 1338)											
rsID	Cluster	chr:pos_nonEA_EA	*β* (SE) *P*	*Q* *P* (het)	*I* ^2^	*β* (SE) *P* (ALSP)	*β* (SE) *P* (UKBSF)	Location	Lead SNP locus (or distance to genes within 200 kb, in bp)	Genes in this cluster	*β* (SE) *P* (CC)^a^
rs150387260	8:53704895-53704896	8:53704896_G_A	0.405 (0.084) *p =* 1.65e−06	0.025 *p =* 0.875	0	0.397 (0.098) *p =* 4.98e−05	0.428 (0.169) *p =* 0.011	intergenic	*RB1CC1* (dist = 77,870), *NPBWR1* (dist = 147,572)	.	0.720 (0.336) *p =* 0.032

The top panel shows hits *p <* 1e−06 from the meta-analysis of all ALSPAC mothers (*N* = 5461) and UKBS females (*N* = 1338): ‘Meta-analysis 1’. The lower panel shows a meta-analysis restricted to females with DNA extracted from white cells (3405 ALSPAC mothers, 1338 UKBS females). In Meta-analysis 2, there were no associations at *p <* 1e−06, so instead the next strongest association is shown

*Abbreviations: rsID* SNP identifier; *chr:pos_nonEA_EA* chromosome, position, non-effect allele, effect allele; *β* regression coefficient (additive model); *SE* standard error; *P p* value; *Q* Cochran’s Q statistic; *bp* base pairs; *P* (het) *p* value for heterogeneity; *I*^*2*^ statistic for heterogeneity; *P* (ALSP) *p* value in ALSPAC analysis contributing to meta-analysis (*N* = 5461 or *N* = 3405); *P* (UKBSF) *p* value in UKBS females analysis; *P (CC) P* (cell-proportion adjusted version of meta-analysis)

^a^NB large drop in number of ALSPAC mothers after cell-proportion adjustment (see Table 1)

#### Meta-analysis 1: all adult females (*N* = 6799)

No SNP passed the genome-wide significance threshold of *p <* 5e−08. The top panel of Table 2 describes two top loci (*p <* 1e−06) identified from the main meta-analysis of all adult females: these included an intergenic locus on chr13 (lead SNP rs12873707, *β* [SE] 0.159 [0.030], *p =* 9.27e−08, *I*^2^ = 0) and a locus on chr17 (lead SNP rs709591, *β* [SE] − 0.084 (0.016) *p =* 1.54e−07, *I*^2^ = 0). A list of all SNPs associated at *p* < 1e−06 in these loci is given in Additional file 3: Table S6. Using summary statistics for all SNPs included in this meta-analysis, we used LD score regression to calculate the estimated total observed scale heritability as 0.052 [SE = 0.079].

Regional association plots (see panels A and B of Fig. 3) show the LD structure at these loci. Panel A shows that there are no nearby SNPs in LD with the lead chr13 variant. Panel B shows a large region of LD at the chr17 locus, spanning the genes *PSMD3*, *CSF3*, and *MED24*. SNPs in this region are associated with neutrophil count [27]; given this fact, and since cellular heterogeneity affects mtDNA CN [22, 55, 56], this locus was assessed in more detail, by extracting the SNP from all study groups (Table 3). Despite differences in the proportion of participants with information that enabled adjustment for cell type, there was consistency across study groups with each showing an approximate 50% attenuation of the effect size with adjustment for cell proportions.

**Table 3:** General concordance of association at rs709591, before adjustment for cell proportions

Cohort	Group	Subgroup	*P*	Info score (imputation quality)	*β*	SE	*N*
Unadjusted							
ALSPAC	Mothers	All	1.05E−06	0.998	− 0.086	0.018	5461
		White cell samples	6.26E−04	0.998	− 0.081	0.024	3405
	Children^b^	All	0.524	0.998	− 0.015	0.024	3647
	Neonates^b^	All	1.36E−04	0.998	− 0.119	0.031	2102
UKBS	All	Females	0.056	0.999	− 0.073	0.038	1338
		Males	0.025	0.999	− 0.090	0.040	1333
Cell-proportion adjusted (see Table 1 for covariates)							
ALSPAC^a^	Mothers	All	0.829	0.997	0.012	0.057	546
		White cell samples	0.774	0.999	0.024	0.085	301
	Children^b^	All	0.702	0.998	0.019	0.051	606
	Neonates^b^	All	0.661	1.000	− 0.08	0.18	82
UKBS	All	Females	0.278	0.999	− 0.044	0.041	1138
		Males	0.241	0.999	− 0.049	0.042	1122

^a^NB large drop in power for ALSPAC in cell proportion adjusted analyses (see Table 1)

^b^Some children in these analyses are related to ALSPAC mothers, however, in *N* = 2833 unrelated ALSPAC mothers, this association persisted (*β* [SE] − 0.120 [0.024], *p =* 4.01e−07)

#### Meta-analysis 2: adult females with white cell-extracted DNA (*N* = 4743)

The bottom panel of Table 2 gives details of the strongest association in the meta-analysis restricted to ALSPAC mothers with DNA extracted from white cells (‘Meta-analysis 2’). The locus associated with mtDNA CN (*p <* 1e−06) was rs150387260, an intergenic variant (*β* [SE] 0.405 [0.084], *p =* 1.65e−06, *I*^2^ = 0, see also Additional file 3: Table S7). A regional association plot (panel C of Fig. 3) shows that few neighbouring SNPs are in substantial LD with this SNP. The nearest gene was RB1 Inducible Coiled-Coil 1 (*RB1CC1*), 78 kb upstream. The product of this tumour suppressor gene is implicated in cell growth, migration, proliferation, apoptosis, and autophagy [57]. *RB1CC1* deletions are associated with increased numbers of mitochondria in haematopoietic stem cells [58] and mice [59] and with breast cancer in humans [60].

#### Effect of adjusting for cell proportions in these two meta-analyses

In Meta-analysis 1 (all adult females, regardless of DNA source), point estimates attenuated after cell proportion adjustment, although the reduced sample size meant that confidence intervals were wide. In contrast, after cell proportion adjustment for the association identified from Meta-analysis 2 (restricted to females with white cell-extracted DNA), confidence intervals were also wide, but the effect estimate was not attenuated (it increased slightly).

### Secondary analyses

Table 4 shows results of GWAS in ALSPAC 6–9-year-olds, neonates, and UKBS males. Only genome-wide-significant loci are shown (for UKBS males, top loci at *p <* 1e−06 are shown, as there were no loci *p <* 5e−08). When comparing effect sizes between any hits in these groups with effects of the same SNPs in the main (adult female) analyses, it should be noted that there are ~ 1600 mother-child duos between ALSPAC mothers included in the main analysis and the 6–9-year-olds/neonates (see the ‘Methods’ section). Results of the associations of SNPs at a known neutrophil count locus (*PSMD3*, *CSF3*, *MED24*) identified in our main analyses in these secondary analysis groups are discussed above (see the ‘Main analysis’ section).

**Table 4:** Lead SNPs in secondary analyses

ALSPAC 6–9-year-olds (*N* = 3647)										
rsID	Cluster	chr:pos_nonEA_EA	*β* (SE) *P*	*β* (SE) *P* (proxy)	Location	Nearby genes	Lead SNP locus (or distance to genes within 200 kb, in bp)	*β* (SE) *P* (CC)^a^	*β* (SE) *P* (MA1)	*β* (SE) *P* (MA2)
rs139045492	20:22836201-22836202	20:22836202_G_A	− 0.695 (0.119) *p =* 6.35E−09	–	Intergenic	*.*	*SSTR4* (dist = 179,855)	− 0.489 (0.240) *p =* 0.042	0.080 (0.078) *p =* 0.306	− 0.005 (0.097) *p =* 0.956
rs144874692	3:175689046-175689047	3:175689047_G_A	− 0.632 (0.112) *p =* 1.78E−08	− 0.328 (0.105) *p =* 0.002	Intergenic	*.*	*NAALADL2* (dist = 165,619)	− 0.024 (0.263) *p =* 0.926	Proxy: 0.00 (0.075) *p =* 1.00	Proxy: 0.016 (0.094) *p =* 0.861
ALSPAC neonates (*N* = 2102)										
rsID	Cluster	chr:pos_nonEA_EA	*β* (SE) *P*	*β* (SE) *P* (Proxy)	Location	Nearby genes	Lead SNP locus (or distance to genes within 200 kb, in bp)	*β* (SE) *P* (CC)^a^	*β* (SE) *P* (MA1)	*β* (SE) *P* (MA2)
rs10424198	19:17354824-17461934	19:17409671_C_T	− 0.262 (0.034) *p =* 1.40E−14	–	Intronic	*ABHD8*, *ANKLE1*, *ANO8*, *BABAM1*, *DDA1*, *GTPBP3*, *MRPL34*, *NR2F6*, *PLVAP*, *USHBP1*	*ABHD8*	− 0.326 (0.193) *p =* 0.095	− 0.010 (0.017) *p =* 0.556	0.002 (0.022) *p =* 0.911
UKBS (males) (*N* = 1333)										
rsID	Cluster	chr:pos_nonEA_EA	*β* (SE) *P*	*β* (SE) *P* (Proxy)	Location	Nearby genes	Lead SNP locus (or distance to genes within 200 kb, in bp)	*β* (SE) *P* (CC)	*β* (SE) *P* (MA1)	*β* (SE) *P* (MA2)
rs11687359	2:1969703-1969704	2:1969704_G_A	0.401 (0.078) *p =* 2.94e−07	–	Intronic	*MYT1L*	*MYT1L*	0.362 (0.080) *p =* 6.52e−06	0.006 (0.032) *p =* 0.845	0.043 (0.041) *p =* 0.286
rs13070764	3:73309902-73310337	3:73309903_G_A	− 0.497 (0.099) *p =* 5.84e−07	–	Intergenic	*.*	*PPP4R2* (dist = 194,892), *PDZRN3* (dist = 121,678)	− 0.509 (0.100) *p =* 4.15e−07	0.100 (0.043) *p =* 0.020	0.098 (0.060) *p =* 0.101

*Abbreviations: rsID* SNP identifier; *chr:pos_nonEA_EA* chromosome, position, non-effect allele, effect allele; *bp* base pairs; *β* regression coefficient (additive model); *SE* standard error; *P p* value; *P (Proxy) p* value (for a proxy SNP, rs144928561, *r*^2^ = 0.29); *P (CC) p* value (cell-proportion adjusted analysis); *P (MA1) p*value (‘Meta-analysis 1’, 6799 adult females); *p value (MA2) P* (‘Meta-analysis 2’, 4743 females with DNA from white cells)

^a^NB large drop in power for ALSPAC mothers (see Table 1)

#### Associations in ALSPAC 6–9-year-olds

There were two genome-wide significant, intergenic loci in 6–9-year-olds (rs139045492 on chr20, *β* [SE] − 0.695 [0.119], *p =* 6.35e−09 and rs144874692 on chr3, *β* [SE] − 0.632 [0.112] *p =* 1.78e−08). The rs139045492 association persisted after cell proportion adjustment. The SNP rs139045492 was not directionally concordantly (or statistically significantly) associated with mtDNA CN in either of the adult female main meta-analyses. The other association (rs144874692), which attenuated after cell-proportion adjustment, was not available in the main analysis. Therefore, a proxy (rs144928561, *r*^2^ = 0.29, *β* [SE] − 0.328 [0.105], *p =* 0.002 in ALSPAC 6–9-year-olds) was used to explore whether there was any evidence of it being associated with mtDNA CN in the main analyses. This proxy SNP was not directionally concordantly associated in either main meta-analysis. Manhattan/QQ plots are available in Additional file 2: Figure S7. A list of all SNPs at *p <* 1e−06 in the 6–9-year-olds is available in Additional file 3: Table S8.

#### Associations in ALSPAC neonates

The locus most strongly associated with mtDNA CN in the ALSPAC neonates was at chromosome 19, in *ABHD8* (abhydrolase-domain containing 8) (lead SNP: rs10424198, *β* [SE] − 0.262 (0.034), *p =* 1.40e−14). Sixteen SNPs were identified at genome-wide significance within *BABAM1*, *ANKLE1*, *ABHD8*, and *MRPL34*. Whilst the standard error for this SNP was large after cell-composition adjustment, this latter analysis was in a tiny sample (*N* = 82), and yet still the point estimate remained broadly consistent (cell-proportion adjusted *β* [SE] − 0.326 [0.193], *p =* 0.095). However, there was no evidence of a concordant association in either meta-analysis from the main analyses. Manhattan/QQ plots are available in Additional file 2: Figure S8. A list of SNPs associated with mtDNA CN at *p <* 1e−06 in neonates is available in Additional file 3: Table S9.

#### Associations in UKBS (males)

No SNPs were associated at *p <* 5e−08 in UKBS males. Two SNPs were associated at *p <* 1e−06: an intronic SNP in *MYT1L* (Myelin Transcription Factor 1 Like, *β* (SE) 0.401 (0.078), *p =* 2.94e−07) and two intergenic SNPs 121 kb upstream of *PDZRN3* (PDZ Domain Containing Ring Finger 3, lead SNP *β* [SE] − 0.497 [0.099], *p =* 5.84e−07). Whilst these SNPs survived cell proportion adjustment, neither locus showed evidence of an association concordant in terms of direction and magnitude in either main meta-analysis. Manhattan and QQ plots for this analysis are available in Additional file 2: Figure S3. A list of SNPs at *p <* 1e−06 in UKBS males is available in Additional file 3: Table S10.

### Look-up of top loci from a recent large GWAS of in silico mtDNA CN

**Fig. 4:**
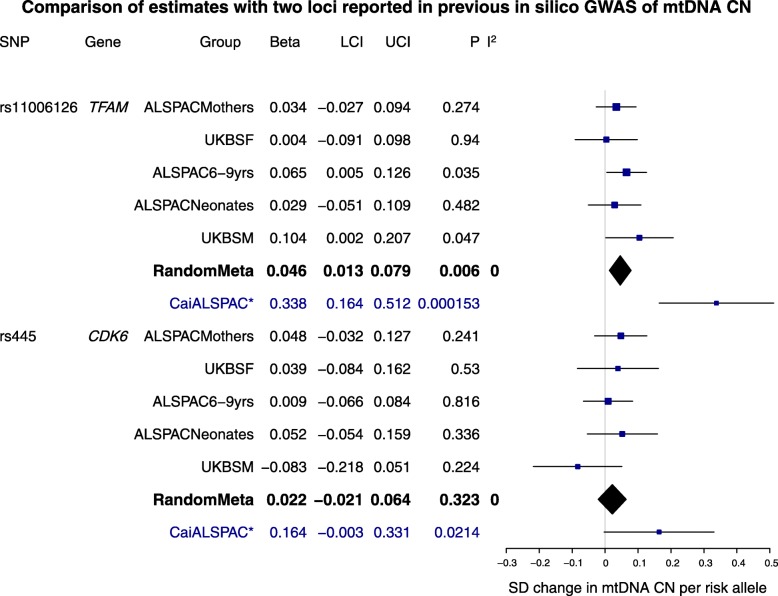
Extraction and meta-analysis of two top loci from Cai et al. [17] from study groups in this GWAS. SNPs for two loci identified in a GWAS of in silico estimated mtDNA CN were extracted from each of the study groups in this cohort (NB: a smaller subset of 2833 ALSPAC mothers were used, since there were mother-child duos present between the original study group of 5461 and the two groups of ALSPAC children) total *N* = 11,253. Columns: SNP = rsID; gene = gene name; group = study group, beta = effect size; LCI/UCI = 95% confidence interval (lower, then upper bound); P = *p* value; and I^2^ = *I*^*2*^ metric for heterogeneity. Meta-analyses were by random-effects and are shown as black diamonds. For reference, the ALSPAC estimate from Cai et al. [17] is shown for each locus. This replication group included ALSPAC 6–9-year-olds (with mtDNA CN assayed from sequence data). Betas had to be harmonised, as those in Cai et al. [17] were given as SD change in mtDNA CN per SD increase in genotype. SD of genotype was estimated from allele frequencies provided for the cohort by Cai et al. [17] given as 0.342 for rs445 and 0.169 for rs11006126 (in the supplement of this paper). SDs were then calculated as √(2 × (1-MAF) × MAF) (evaluating to 0.53 and 0.67 for rs11006126 and rs445, respectively). Betas and standard errors were then transformed from those given in Table 1 of Cai et al. to a ‘per risk allele’ scale, by multiplying the given beta by (1/the estimated SD), i.e. rs11006126 = 0.179 × (1/0.53); rs445 = 0.110 × (1/0.67). CaiALSPAC = result from Cai et al. [17]. UKBSF/UKBSM = females and males in UKBS cohort

The results of a look-up of two loci (rs11006126 and rs445) that are strongly associated with mtDNA CN measured in silico [17] are presented in Fig. 4. Estimates for these SNPs were extracted from and meta-analysed across all study groups (after removing ALSPAC mother-offspring pairs; total *N* for these meta-analyses = 11,253, see also the ‘Genotype data’ section). The SNP rs11006126 was associated with mtDNA CN across our study groups (*β* [SE] 0.046 [0.017], *p =* 0.007), but the effect size was considerably (~ 1/7 of the size) smaller than that observed in the discovery and replication cohorts of the in silico GWAS. There was no evidence of replication of rs445 across our study groups (*β* [SE] 0.021 [0.022], *p =* 0.328) compared with discovery and replication result from the in silico GWAS.

## Discussion

We conducted the largest ever GWAS of directly assayed mtDNA CN, in two population-based cohorts. Although diversity between our study groups prevented us from conceptualising a traditional discovery and replication model, we considered our study as hypothesis generating and meta-analysed the two most comparable groups (ALSPAC mothers and UKBS females) as our main analyses and other subgroups as opportunistic, secondary analyses.

There were no genome-wide significant hits in the meta-analysis of adult females, but SNPs at a known neutrophil (and other leucocytes [61]) count locus (*PSMD3*, *CSF3*, *MED24*) [62] were associated with mtDNA CN at *p* ~ 2e−07. There is extended LD across these genes (see Fig. 3); although *CSF3* (alias granulocyte colony-stimulating factor) may be the most likely biological candidate [63], a previous study in Japanese individuals reported that the neutrophil count associated SNP was most associated with *PSMD3* expression [62]. It is established that cellular heterogeneity is related to mtDNA CN [22], and one of the strengths of this study is that we were able to control for this. This chr17 locus showed notable consistency of effect sizes across several study groups (UKBS males, UKBS females, ALSPAC neonates), corresponding to a ~ 0.08 reduction in SD units of log mtDNA CN per risk allele. However, there was notable (~ 50%) attenuation with adjustment for white cell proportions. Thus, a key finding of this study is the importance of undertaking GWAS of directly assayed mtDNA CN in sample sizes that are much larger and that also have measures of white cell proportions. Ideally, these studies should also be sufficiently large to enable exploration of any possible variation in nuclear genetic control of mtDNA copy number variation by sex and age.

Secondary analyses in ALSPAC children (6–9 years) and ALSPAC neonates and UKBS males revealed three genome-wide significant hits: two intergenic loci in ALSPAC 6–9-year-olds and a region containing *ABHD8* (abhydrolase-domain containing-8) in ALSPAC neonates. None of the associations we observed at this locus in ALSPAC neonates were at all evident in the main meta-analysis of adult females. We only identified one published association of SNPs within *ABHD8* with a disease trait (breast cancer) in the literature [64], although it is notable that this gene lies in head-to-head orientation with mitochondrial ribosomal protein L34, *MRPL34*. Another mitochondrial ribosomal protein, *MRPL37*, has previously been found to associate with mtDNA CN, which [20] raises the possibility that the *BABAM1-ANKLE1-ABHD8-MRPL34* locus might be a true signal, despite the lack of evidence of effect in our main analyses. Mitochondrial ribosomal proteins are encoded by nuclear DNA, synthesised on cytoplasmic ribosomes, then imported into mitochondria, where they facilitate the translation of mitochondrial mRNA, in conjunction with the two mitochondrially encoded rRNAs [65]. Whilst there are no candidate disorders for *MRPL34*, other diseases, such as Parkinson’s disease (previously linked to reduced mtDNA CN [66]), are related to other mitochondrial ribosomal proteins [20, 65, 67]. This locus also included an additional neighbouring gene, GTP Binding Protein 3 (Mitochondrial) (*GTPBP3*), associated with mtDNA CN at *p* ~ 5e−07; rare mutations in this gene are known to cause a Leigh syndrome-like disorder [68]. However, it is clear that independent replication will be needed in order to confirm these associations, since our current results are in a small sample from our secondary analyses only, and could be attributable to ‘winner’s curse’ [69]. In addition, if such an association were to replicate in future GWAS, careful fine-mapping would be needed in order to further refine the signal and suggest putative causal genes out of those found to be associated.

The identification of the cell-count associated locus (lead SNP in *MED24*) could suggest that some loci, such as this chr17 locus, may be related to mtDNA CN only via their association with cell composition. It is noteworthy that the meta-analysis in which this locus was identified included ALSPAC mothers with DNA extracted from both white cells and whole blood: it is possible that combining individuals with DNA prepared from multiple sources could lead to preferential detection of loci associated with mtDNA CN predominantly via their association with cellular heterogeneity. When results from participants with more similar DNA sources are pooled, the power to detect loci associated with mtDNA CN *independently* of cell proportions may increase: we postulate that the *BABAM1-ANKLE1-ABHD8-MRPL34* locus might be such a ‘cell-composition independent’ locus (although as noted above, this needs further exploration). For loci in this latter category, controlling for cell proportions may improve the signal-to-noise ratio of observed associations, and failure to control for cellular proportions may act akin to measurement error. Future work should seek to assert whether associations of known neutrophil loci with mtDNA CN are entirely due to cell composition in DNA samples or whether these loci are detected because mtDNA CN is causally related to leucocyte count [70]. Ideally, such studies would use directly assayed neutrophil data, as opposed to estimates of cellular composition. In addition, it might be useful to control associations for platelet count, since platelets are anucleate, but mitochondria-rich [71, 72]. However, we acknowledge that in adjusting for cellular composition, it is possible that we could induce bias, if mtDNA CN plays a causal role in determining cell counts. It is for this reason that we chose to present models adjusted and unadjusted for cellular composition in our work, and we compare the results throughout.

Beyond the possibility of true underlying genetic heterogeneity between our cohorts, several other technical factors may have limited their comparability. We believe that population stratification is unlikely to be a problem, as analyses controlled for principal components, and the correlation of MAFs for tops hits was high (see Additional file 2 Figure S9). Another technical difference between study groups is the DNA extraction method used. DNA extraction method has a considerable effect on mtDNA CN assay [73] and similar qPCR assays, including that of telomere length [74]. However, it is difficult to assess the extent to which DNA extraction method may have affected the performance of the mtDNA CN assay, since extraction method in ALSPAC was also related to age at DNA sampling, and it is possible that the genetic regulation of mtDNA CN varies across the life course.

When we combined all our study groups and looked at whether two hits from a previous in silico GWAS were replicated, we found some evidence for one (a SNP in mitochondrial transcription factor A (*TFAM*)), but not the other (*CDK6*) [17]. Notably, the previously reported SNP for the CDK6 locus (rs445) has also been shown to be associated with leucocyte counts [61]. *TFAM* is known as ‘a key activator of mitochondrial transcription’ and is involved in ‘promoter recognition by the mitochondrial RNA polymerase’ [16], yet despite the comparable sample sizes between our (*N* = 11,253) and the previous in silico (*N* = 10,442 discovery and *N* = 1753 replication) analyses, we observed a considerably smaller effect size for the *TFAM* hit (~ 1/7 the size of the replication effect from the previous study, despite partial overlap in participants between the previously published result and our sample). We postulate that this might flag an important limitation in our work, namely that of measurement error. If a substantial component of our assayed mtDNA CNs includes non-differential measurement error, we would expect to see attenuation of effect sizes in our results. This is possible in qPCR assays; indeed, although we demonstrated correlations in relative mtDNA CNs in a validation analysis, we saw some suggestion of non-linearity. Whilst our attempted replication of the previous in silico GWAS hit had 100% power to detect effect sizes of 0.338 SD units (for a SNP with a MAF of 0.169, i.e. the *TFAM* SNP), this power calculation will be overoptimistic if our mtDNA CN assays are affected by measurement error. Therefore, we might suggest that in silico measurement of mtDNA CN may have advantages over the directly assayed method in this instance.

## Conclusion

In conclusion, we confirm an association of *TFAM* with mtDNA CN, and after performing a range of hypothesis-generating GWAS in diverse study groups, we present several putative regulators of mtDNA CN that will require further follow-up. However, we generally observed poor concordance across study groups. Overall, our main conclusion is that here we find no strong evidence to support our primary hypothesis of common loci regulating mtDNA CN in the study groups used here. We assess and discuss the possible implications of cellular heterogeneity on our results and present the directly assayed mtDNA CN assay as another example of a qPCR assay that may be subject to measurement error. These findings should be considered as possible power-limiting factors in GWAS studying the genomic regulation of mtDNA CN. Nonetheless, we believe that to fully understand nuclear genomic control of mtDNA CN variation, it is necessary to conduct GWAS of directly assayed mtDNA CN. Thus, our work (the largest GWAS to date) makes an important contribution in terms of future requirements to gain this knowledge, which is necessary for fuller understanding of the biology and potential clinical impact of subtle variation in mtDNA CN.

## Additional files

Additional file 1: Appendices 1-3. (PDF 343 kb)

Additional file 2: Supplementary Figures. (PDF 1250 kb)

Additional file 3: Supplementary Tables. (XLSX 42 kb)
