# Supplementary material for: A genome-wide association study of mitochondrial DNA copy number in two population-based cohorts
Source: Hum Genomics. 2019 Jan 31;13:6. doi: 10.1186/s40246-018-0190-2 (PMC6357493; doi:10.1186/s40246-018-0190-2)
Supplement: Supplementary file 1 — Appendices 1-3. (PDF 343 kb) [file 40246_2018_190_MOESM1_ESM.pdf]

Additional file 1

A genome-wide association study of mitochondrial DNA copy number in two population-based cohorts

Anna L. Guyatt, BSc MBChB PhD[1,2,7] Rebecca R. Brennan, BSc MRes PhD,[3,7] Kimberley Burrows, BSc PhD[1,2] Philip A. I. Guthrie, MSc PhD[2] Raimondo Ascione, MD MSc ChM FETCS FRCS[4] Susan Ring, BSc PhD[1,2] Tom R. Gaunt, BSc PhD[1,2] Angela Pyle, BSc PhD[3,5] Heather J. Cordell, BA, MSc, DPhil[5] Debbie A. Lawlor, MSc MBChB PhD MPH MRCGP MFPHM[1,2] Patrick Chinnery, MBBS PhD FRCPath FRCP FMedSci[6] Gavin Hudson, BSc PhD[3,8] Santiago Rodriguez, BSc MSc PhD[1,2,8]*

1. MRC Integrative Epidemiology Unit at the University of Bristol, Bristol, BS8 2BN, UK.
2. Population Health Sciences, Bristol Medical School, University of Bristol, Bristol, UK.
3. Wellcome Centre for Mitochondrial Research, Newcastle University, Newcastle, UK.
4. Bristol Heart Institute, Translational Health Sciences, Bristol Medical School, University of Bristol, Bristol, UK.
5. Institute of Genetic Medicine, Newcastle University, Newcastle, UK
6. Department of Clinical Neurosciences and MRC Mitochondrial Biology Unit, University of Cambridge, Cambridge, UK
7. These authors contributed equally to this work
8. These authors contributed equally to this work

Email: *santiago.rodriguez@bristol.ac.uk

Telephone: +44 117 331 0133

Appendix 1: Genotyping quality control in ALSPAC and UKBS

See **Table I** for summary of quality control.

ALSPAC

Acknowledgements for genotyping and imputation in ALSPAC: Dr Gibran Hemani (University of Bristol), Dr George McMahon (University of Bristol), Professor Jonathan Marchini (University of Oxford).

ALSPAC Mothers were genotyped on the Illumina Human660W-Quad array at the Centre Nationale du Génotypage (CNG). ALSPAC children were genotyped with the Illumina HumanHap550-Quad array, by 23andme, the Wellcome Trust Sanger Institute, Cambridge, UK, and the Laboratory Corporation of America, Burlington, NC, US. Genotypes were called using Illumina GenomeStudio, and quality control (QC) was performed using PLINK v1.07.

19930 individuals entered the quality control pipeline, including 526,688 SNPs in mothers, and 500,527 SNPs in children. SNP-wise filters were as follows: call rate 0.05, HWE p<5e-07, MAF filter 0.01. Sample-wise filters included individual call rate (0.03 [Children] and 0.05 [Mothers]), and outliers for autosomal heterozygosity. In addition, samples with indeterminate X chromosome heterozygosity were removed, (and pseudoautosomal regions have been removed). Cryptic relatedness filters were 0.125 for mothers, and 0.1 for children, according to PIHAT values calculated by PLINK. Samples were removed if they clustered outside of the CEU HapMap2 population, using multidimensional scaling of genome-wide IBS pairwise distance.

After these QC steps, 9,115 children [500,527 SNPs] and 9,048 mothers [526,688 SNPs] were retained. Amongst these 18163 individuals, there were 477,482 SNPs in common. Further filtering in this set included the removal of SNPs with missingness due to poor quality (>0.01, n=11,396), and 321 people due to possible ID mismatches. The final dataset therefore consisted of 17,842 subjects (6,305 duos) and 465,740 SNPs (a final 112 were removed during liftOver, and 234 because they were no longer in Hardy-Weinberg Equilibrium after combination).

Haplotypes were estimated using ShapeIT (v2.r644), and relatedness was utilised during phasing. Phased haplotypes were imputed to the Haplotype Reference Consortium (HRC) panel of approximately 31,000 phased whole genomes. The HRC panel itself was phased using ShapeIt (v2), with imputation performed using IMPUTE (v3).

UKBS

The UKBS cohort was genotyped using the Illumina 1.2M Duo platform (Illumina San Diego California USA). Raw genotype data for the UK National Blood Service (UKBS) were downloaded from the European Genotype Archive (http://www.ebi.ac.uk/ega). Genotype data were filtered on missing data <5%, genotype missing data <5%, MAF <1% and HWE 1.00e-06. After imputation to HRC v1.0 release, SNPs were filtered for MAF >0.01 and info >0.8.

Quality control was performed using PLINK v1.90b3p 64-bit (2 Aug 2014). From 2682 individuals (1336 males and 1346 females) with mitochondrial copy number and genotype data, 11 people were removed due to missing data <5%, 4249

variants were removed due to missing genotype data <5%, 187,314 variants were removed due to minor allele threshold at 1% and 12,344 variants were removed due to Hardy-Weinberg exact test threshold of 1.00e-06. 948,779 variants and 2671 people (1333 males and 1338 females) passed QC and total genotyping rate was 0.997998.

Following QC, samples had an average rate of 0.0016 (±0.0048 s.d) missingness data. Clustering analysis was performed and one, single cluster was identified. Identical-by-descent (IBD) analysis was performed across default size, 500kbp regions, at the standard threshold 0.85, then 0.2 and then without a threshold. No individuals were IBD at any threshold.

Autosomes were imputed by the Sanger Imputation Server (https://imputation.sanger.ac.uk/). Pre-phasing and imputation was performed by EAGLE2 (v2.0.5) and PBWT against the Haplotype Reference Consortium (release 1.0) (http://www.haplotype-reference-consortium.org/). 39,235,157 variants were imputed. 19,210,271 variants were removed due to imputation score <0.8 and 12,583,396 variants (12,654,900 males only and 12,651,394 females only) were removed due to MAF threshold <1%. 7,441,490 variants (7,369,986 males only and 7,373,492 females only) remained.

Table I Summary of quality control measures

<table>
<tr><th>Stage</th><th colspan="2">Metric</th><th colspan="3">Cohort</th></tr>
<tr><th></th><th colspan="2"></th><th>ALSPAC Mothers</th><th>ALSPAC Children and Neonates</th><th>UKBS</th></tr>
<tr><td rowspan="12">Pre-imputation</td><td colspan="2">Genotyping platform</td><td>Illumina Human660W-Quad</td><td>Illumina HumanHap550-Quad</td><td>Illumina 1.2M Duo</td></tr>
<tr><td colspan="2">Software for QC</td><td>PLINK v1.07</td><td>PLINK v1.07</td><td>PLINK v1.90b3p</td></tr>
<tr><td rowspan="3">SNP-wise filters</td><td>Call rate</td><td>0.05</td><td>0.05</td><td>0.05</td></tr>
<tr><td>HWE</td><td>p<5e-07</td><td>p<5e-07</td><td>P<1e-06</td></tr>
<tr><td>MAF</td><td>0.01</td><td>0.01</td><td>0.01</td></tr>
<tr><td rowspan="5">Sample-wise filters</td><td>Individual call rate</td><td>0.05</td><td>0.03</td><td>0.05</td></tr>
<tr><td>Outliers for autosomal heterozygosity</td><td colspan="2">Removed</td><td>Removed</td></tr>
<tr><td>Samples with indeterminate X chromosome heterozygosity (pseudoautosomal regions removed)</td><td colspan="2">Removed</td><td>Removed</td></tr>
<tr><td>Cryptic relatedness</td><td>IBD>0.125</td><td>IBD>0.1</td><td>IBD>0</td></tr>
<tr><td>Population</td><td colspan="2">Samples removed if clustered outside of CEU HapMap2, using multidimensional scaling</td><td>See Burton et al., 2007</td></tr>
<tr><td rowspan="3">Imputation</td><td colspan="2">Haplotype estimation and phasing</td><td colspan="2">ShapeIT (v2.r644)</td><td>EAGLE2 (v2.0.5)</td></tr>
<tr><td colspan="2">Imputation software</td><td colspan="2">IMPUTE (v3)</td><td>PBWT (Sanger Imputation Server)</td></tr>
<tr><td colspan="2">Imputation panel</td><td colspan="3">Haplotype Reference Consortium (HRC) panel v1.0</td></tr>
<tr><td rowspan="4">Post-imputation</td><td rowspan="2">SNP-wise</td><td>MAF</td><td colspan="3">0.01</td></tr>
<tr><td>Imputation quality</td><td colspan="3">0.8</td></tr>
<tr><td colspan="2"># variants included</td><td>7,360,988</td><td>7,410,776 / 7,361,275</td><td>7,441,490</td></tr>
<tr><td colspan="2"># samples included</td><td>5461</td><td>3647 / 2102</td><td>2671</td></tr>
</table>

Appendix 2: Assay of mitochondrial DNA copy number in ALSPAC and UKBS

ALSPAC

MtDNA CN was measured using a quantitative PCR (qPCR) assay that was optimised in-house. This singleplex assay relates the relative copy number of a mitochondrial gene [bases 317-381 in the D-loop region] to a nuclear reference gene [*B2M*]. For each of the mitochondrial and nuclear gene reactions, a master mix was made using 5μL 2x SensiFAST SYBR No-ROX Kit (Bioline), 250nM of each of the forward and reverse primers, and 0.5μL water. Primer sequences (5'-3') were hmitoF5 CTTCTGGCCACAGCACTTAAAC and hmitoR5 GCTGGTGTTAGGGTTCTTTGTTTT for the mtDNA amplicon, and hB2M_F2 GCTGGGTAGCTCTAAACAATGTATTCA and hB2M_R2 CCATGTACTAACAAATGTCTAAAATGGT for the nuclear DNA amplicon.[60] 1ng of DNA in a 4μL volume (i.e. 0.25ng/μL) was added to each of 6 microplate wells, to which 6μL aliquots of the respective master mixes were added, giving a final reaction volume of 10μL per well. Each of the mitochondrial and nuclear reactions was undertaken in triplicate. Samples were assayed using a Roche LightCycler LC480 and 384-well plates under the following thermocycler conditions: 5 minutes at 95^{0}C (1 cycle), then 45 cycles of: 5 seconds at 95^{0}C→15 seconds at 55^{0}C → 15 seconds at 72^{0}C → 1 second at 78^{0}C (with signal acquisition).

Standard curves were generated from pooled results of 4 DNAs, and were used to calculate reaction efficiencies. These efficiency values were used to adjust raw crossing point (Cp) data. Efficiency-adjusted Cps were then used to calculate mtDNA CN.[61]

In order to control for run-to run variability, 3 calibrator DNAs were run on every plate. The average of the calibrators on each plate were compared to the average of all of the calibrators across all plates, allowing the derivation of a per-plate calibration factor, by which each plate value was multiplied.

8159 mothers (4691 6-9 year olds, 2889 neonates) were originally assayed for mtDNA. After relatedness pruning and exclusion of those without genotype and covariate data, 5461, 3647 and 2102 individuals remained.

UKBS

Mitochondrial DNA was quantified in all 3091 UK National Blood Service (UKBS) samples by multiplex Taqman qPCR amplification (assays undertaken by RB) of the nuclear encoded gene *B2M*, and the mitochondrial genes *MTND1* and *MTND4*, as described previously.[62] All samples were assigned to each run randomly, to minimise run-specific stratification. mtDNA CN is expressed as copies per cell as described previously.[59]

Appendix 3: Presentation of top hits in ALSPAC Mothers and UKBS Females

1. *Associations in ALSPAC Mothers: evidence of the importance of cellular heterogeneity*

All clusters of loci below p<1e-06 are shown in **Supplementary Table 1**. The two panels of this table represent the results of the 'complete' analysis of all mothers (n=5461) and the analysis restricted to mothers with DNA from white cells only (hereafter the 'WC' analysis, n=3405).

Regions of association were clustered into 1Mb chunks using the 'clusterBed' function of 'bedtools'.[64] The lead SNP in each region is shown, along with effect sizes (in Z-scores), standard errors, and P values, for both the main, and cell-proportion adjusted (CC) analyses. Location (according to hg19 coordinates) and annotation information is also given for each locus. If no locus was annotated by the ANNOVAR software, loci within 200kb (after which LD levels tend towards low levels) are reported.[78] Effect sizes, standard errors and P values from two additional analyses are presented: the column 'Beta (SE) P (UKBSF)' refers to the effect size, standard error and P value from the UKBS analysis, restricted to females. The columns 'Beta (SE) P (All)' and 'Beta (SE) P (WC)' are effect sizes, standard errors and P values from the analyses containing all of the ALSPAC mothers, and those restricted to women in whom mtDNA CN was measured in white cells ('WC').

The five loci identified in ALSPAC mothers included two intergenic SNPs, plus three intronic SNPs: one SNP, rs8066582, identified in the complete analysis, is located at 17q12 region, within *PSMD3*. This SNP is known to associate with neutrophil count from previous GWAS.[79]

None of the lead SNPs identified in either of the ALSPAC mothers' analyses (the analysis of all mothers, or the 'WC' analysis) showed evidence of association in UKBS Females: two out of the five SNPs (rs12873707 and rs8066582) had broadly consistent effect sizes and standard errors. After adjustment for cellular proportions, the effect sizes in the analysis of all ALSPAC mothers attenuated, but standard errors were large.

When the analysis was restricted to just those ALSPAC mothers with DNA extracted from white cell pellets, both effect sizes increased in magnitude after adjustment for cellular proportions, and were consistent with the same direction of effect as in the unadjusted analysis (although again, standard errors were large). One 'genome-wide significant' locus was identified (rs75320628 [chr19, *BTBD2*], β(SE)=0.378(0.069), p=4.87e-08). The other locus was an intronic SNP in *GPC6* (rs140305578, p=9.28e-07). *BTBD2* (BTB Domain Containing 2) is localised to cytoplasmic bodies and binds topoisomerase I, and *GPC6* (Glypican 6) has been implicated in control of cell growth and division.[80]

Notes on Supplementary Table 1

The top panel shows the results for ALSPAC Mothers (n=5461), and bottom panel is restricted to just those ALSPAC mothers with DNA extracted from white cells (n=3405, all nested within the total sample size [n=5461]). The final column demonstrates the P value for each of the SNPs associated in the ALSPAC analysis in the other analysis). Abbreviations: rsID=SNP identifier; chr:pos_nonEA_EA=chromosome, position, non-effect allele, effect allele; Beta=regression coefficient (additive model); SE=standard error; P=p value; P (CC)=P (cell-proportion adjusted analysis); P (UKBSF)=P value from UKBS

(females) analysis); P (WC)=P (analysis restricted to white cell extracted samples only), P (All); P (analysis of all ALSPAC mothers). *NB large drop in power for ALSPAC (See **Table 1**)
