# Supplementary material for: A genome-wide association study of mitochondrial DNA copy number in two population-based cohorts
Source: Hum Genomics. 2019 Jan 31;13:6. doi: 10.1186/s40246-018-0190-2 (PMC6357493; doi:10.1186/s40246-018-0190-2)
Supplement: Supplementary file 2 — Supplementary Figures. (PDF 1250 kb) [file 40246_2018_190_MOESM2_ESM.pdf]

SUPPLEMENTARY FIGURES

A genome-wide association study of mitochondrial DNA copy number in two population-based cohorts

Anna L. Guyatt, BSc MBChB PhD[1,2,7] Rebecca R. Brennan, BSc MRes PhD,[3,7] Kimberley Burrows, BSc PhD[1,2] Philip A. I. Guthrie, MSc PhD[2] Raimondo Ascione, MD MSc ChM FETCS FRCS[4] Susan Ring, BSc PhD[1,2] Tom R. Gaunt, BSc PhD[1,2] Angela Pyle, BSc PhD[3,5] Heather J. Cordell, BA, MSc, DPhil[5] Debbie A. Lawlor, MSc MBChB PhD MPH MRCGP MFPHM[1,2] Patrick Chinnery, MBBS PhD FRCPath FRCP FMedSci[6] Gavin Hudson, BSc PhD[3,8] Santiago Rodriguez, BSc MSc PhD[1,2,8]*

1. MRC Integrative Epidemiology Unit at the University of Bristol, Bristol, BS8 2BN, UK.
2. Population Health Sciences, Bristol Medical School, University of Bristol, Bristol, UK.
3. Wellcome Centre for Mitochondrial Research, Newcastle University, Newcastle, UK.
4. Bristol Heart Institute, Translational Health Sciences, Bristol Medical School, University of Bristol, Bristol, UK.
5. Institute of Genetic Medicine, Newcastle University, Newcastle, UK
6. Department of Clinical Neurosciences and MRC Mitochondrial Biology Unit, University of Cambridge, Cambridge, UK
7. These authors contributed equally to this work
8. These authors contributed equally to this work

Email: *santiago.rodriguez@bristol.ac.uk

Telephone: +44 117 331 0133

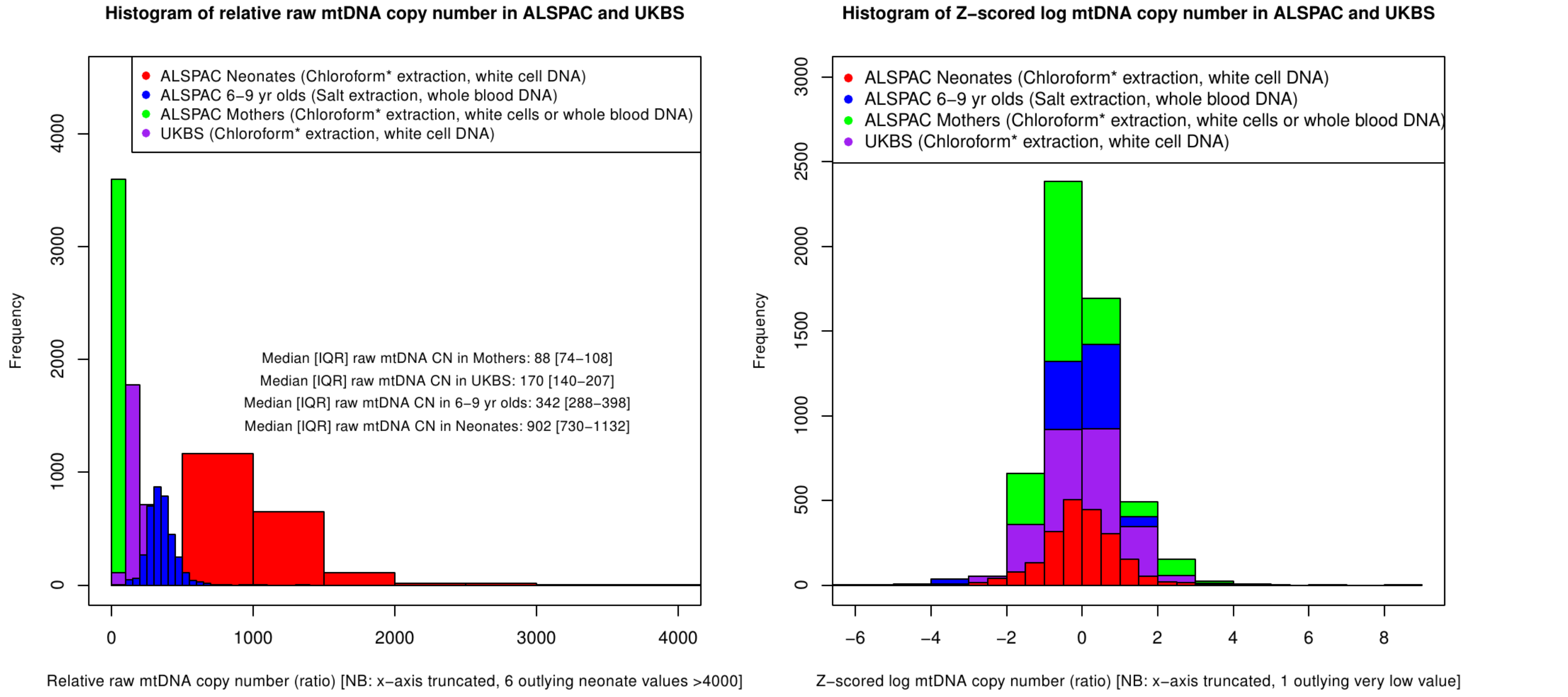

Figure S1. Histograms of raw (left) and z-scores of log transformed mtDNA CN (right) in the cohorts studied. mtDNA CN calculation in ALSPAC was undertaken using efficiency-adjusted crossing points (C_ps) for the nuclear and mitochondrial amplicons, using the equation: mtDNA CN = 2*(2^(nuclear DNA Cp – mitochondrial DNA Cp)). Calculation of mtDNA CN in UKBS was as described previously (see Grady *et* al., 2014, DOI: https://doi.org/10.1371/journal.pone.0114462) *Chloroform extraction methods were different between ALSPAC and UKBS: phenol-chloroform in ALSPAC, guanidine-chloroform in UKBS.

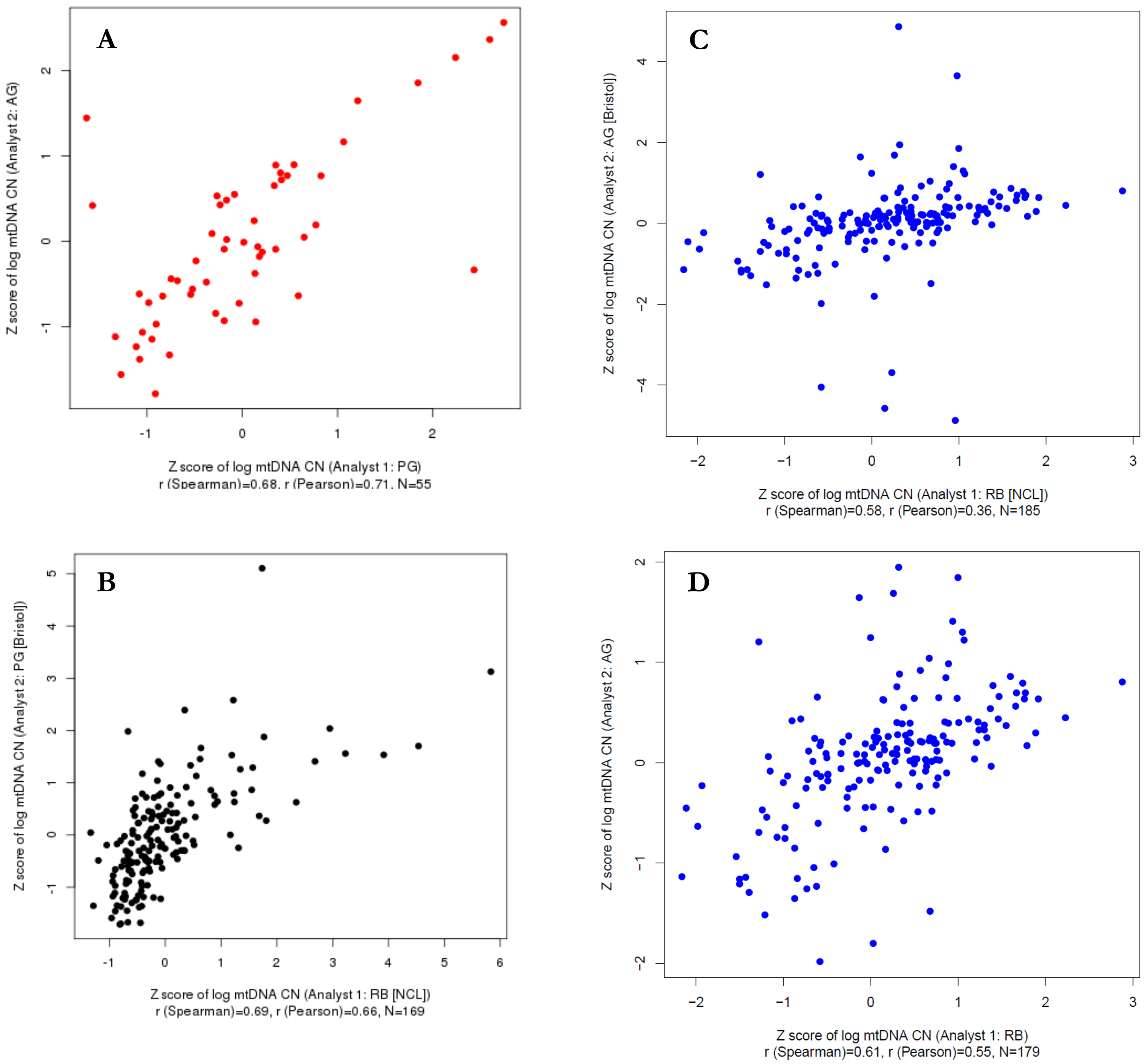

Figure S2. Validation assay involving three analysts. RB (Newcastle) and PG (Bristol) generated all mtDNA CN data used in the GWAS. However, since PG was no longer based in Bristol at the time of the study, AG (Bristol, ALSPAC protocol) and RB (Newcastle, UKBS protocol) carried out some of the validation analyses. Panel A shows the correlations between z scored log mtDNA CN values generated by AG and PG from one plate of ALSPAC DNAs (N=55). Two plates (n=169) of ALSPAC DNA were exchanged, and the counts between PG and RB compared (Panel B). Panel C shows the correlations obtained when two plates of UKBS DNA (n=185) were assayed by AG and RB. The same plot (with 6 outliers +/- 2SDs removed) is shown in Panel D. Generally, there was moderate to good correlations in all comparisons.

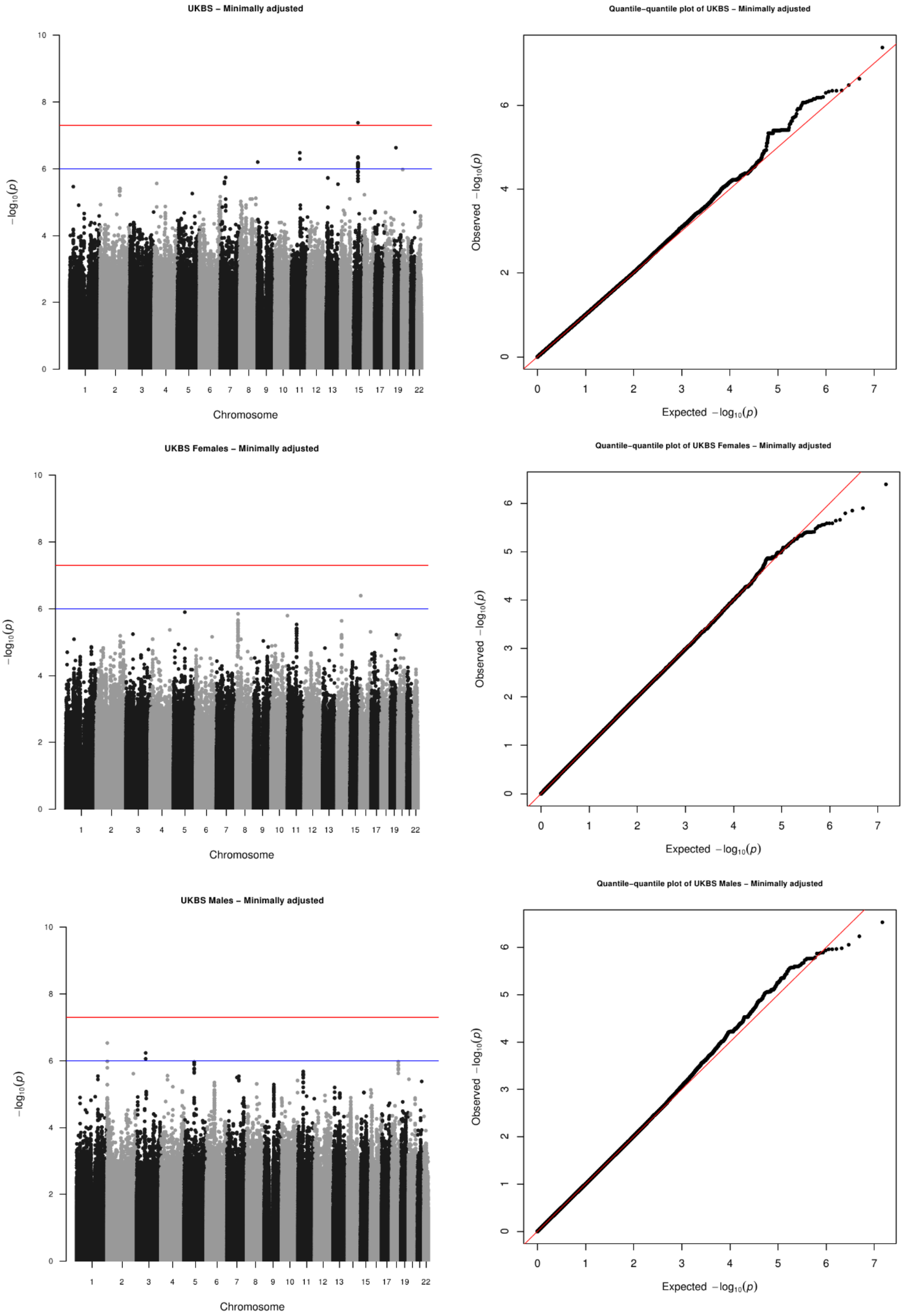

Figure S3. Manhattan (left) / QQ plots (right) for UKBS (all) and UKBS (females), and UKBS (males). Top row=UKBS (all), Middle row=UKBS (females), Bottom row=UKBS (males). □ =1.008, 1.011 and 1.002, respectively.

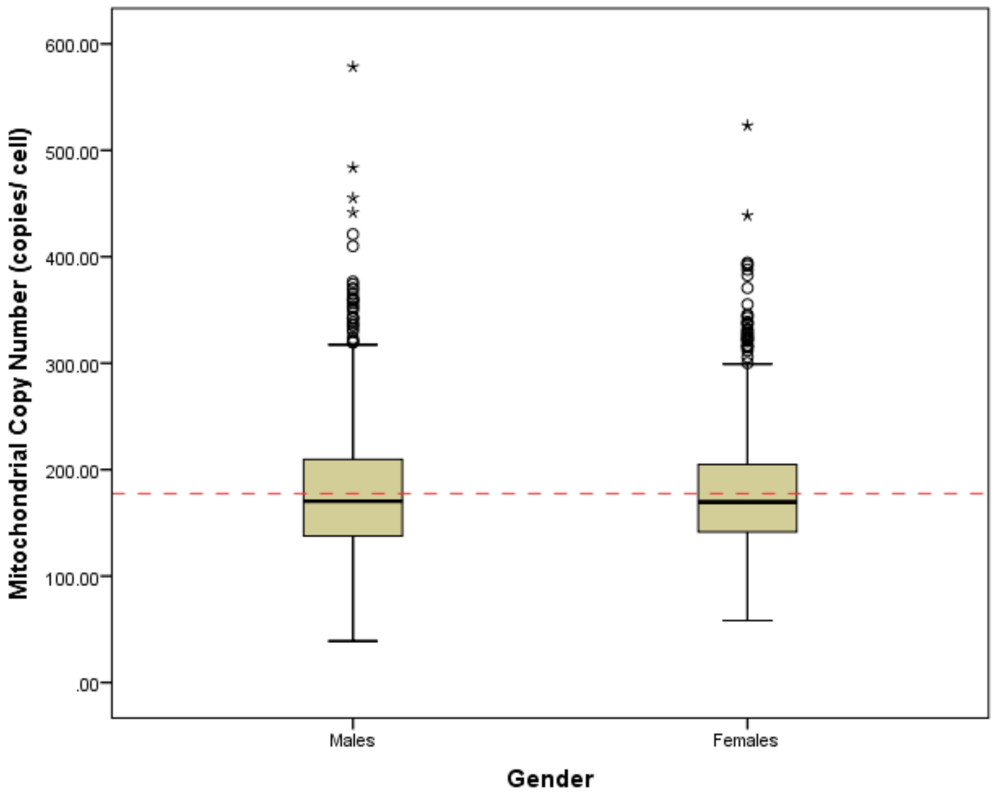

Figure S4. Means of mtDNA CN by sex in UKBS. Males (n=1335): mean (SD)=178.5 (57.0); Females (n=1346)): mean (SEM)=176.6 (52.9). Independent t-test p=0.363, Mann Whitney p=0.722.

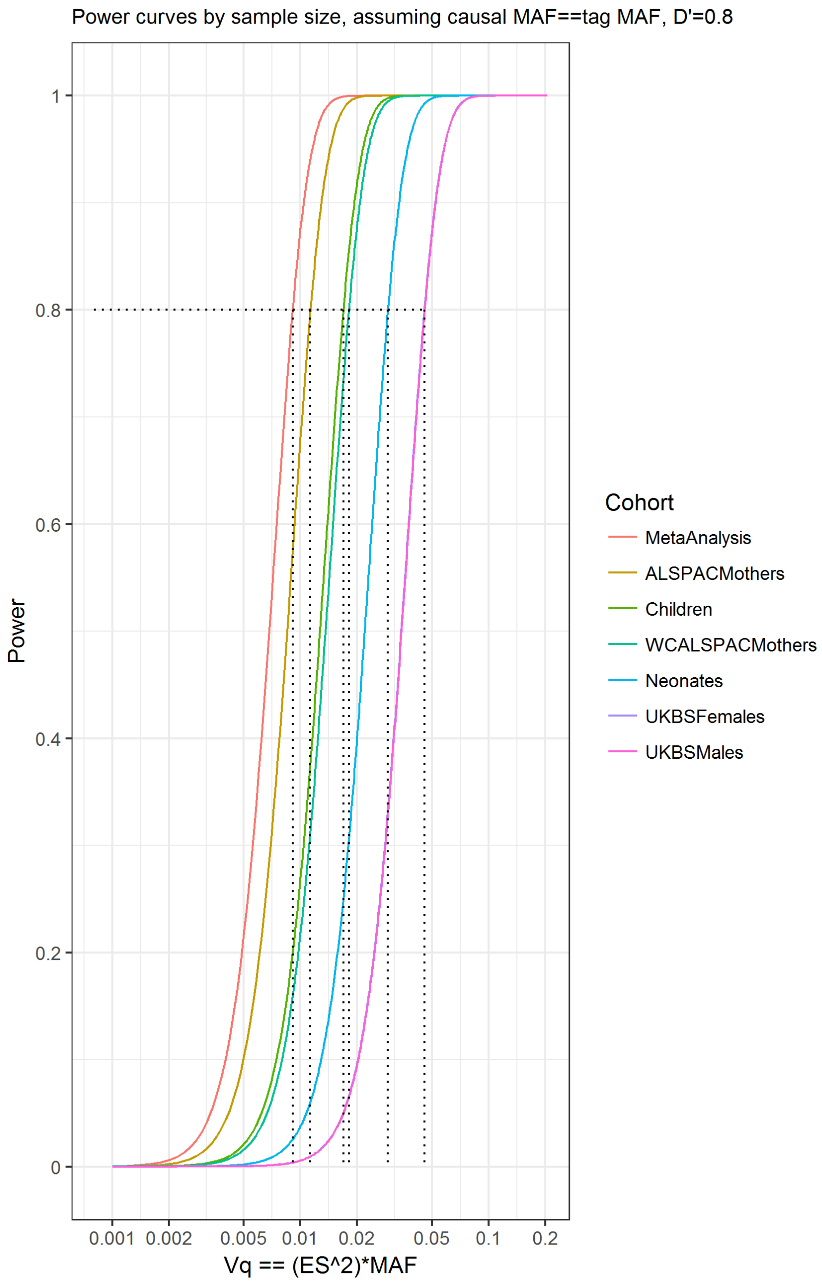

Figure S5. Power curves for a range of values of Vq are shown (where Vq is the total proportion of variance explained by a causal variant). Assuming a phenotype that follows a normal distribution, Vq is equal to the square of the effect size (ES, in standard deviation units) multiplied by the minor allele frequency (MAF). Linkage disequilibrium (LD) is assumed between the causal variant and tag variant at a D' value of 0.8. Different curves are shown for all subgroups used in this paper: MetaAnalysis=ALSPACMothers and UKBS Females; ALSPACMothers (N=5461), Children [6-9 years] (N=3647), ALSPACMothers (white cell samples only, N=3405), Neonates (N=2102), UKBSFemales (N=1338), UKBSMales (N=1333). The black dotted values correspond to the minimum values of Vq that are required for power of 0.8 (i.e. 80%).

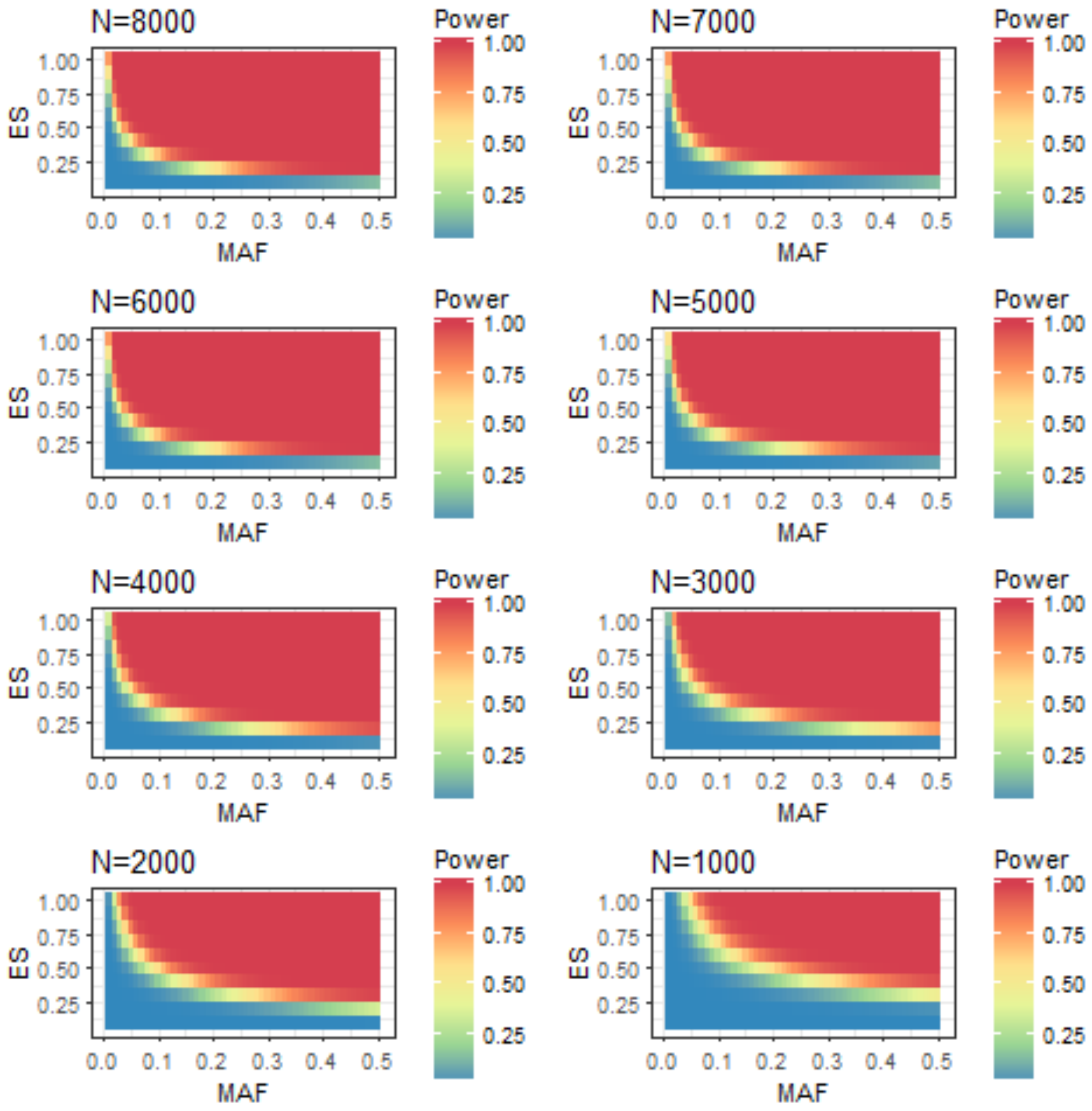

Figure S6. Heat maps of predicted power according to a range of minor allele frequencies (MAF) and standardised effect sizes (ES), stratified by a range of sample sizes encompassing the range of sample sizes in all analysis groups included in this paper. Power improves as a function of increasing sample size, effect size and minor allele frequency. For all plots, LD of 0.8 (D') is assumed between causal variants and tag variants.

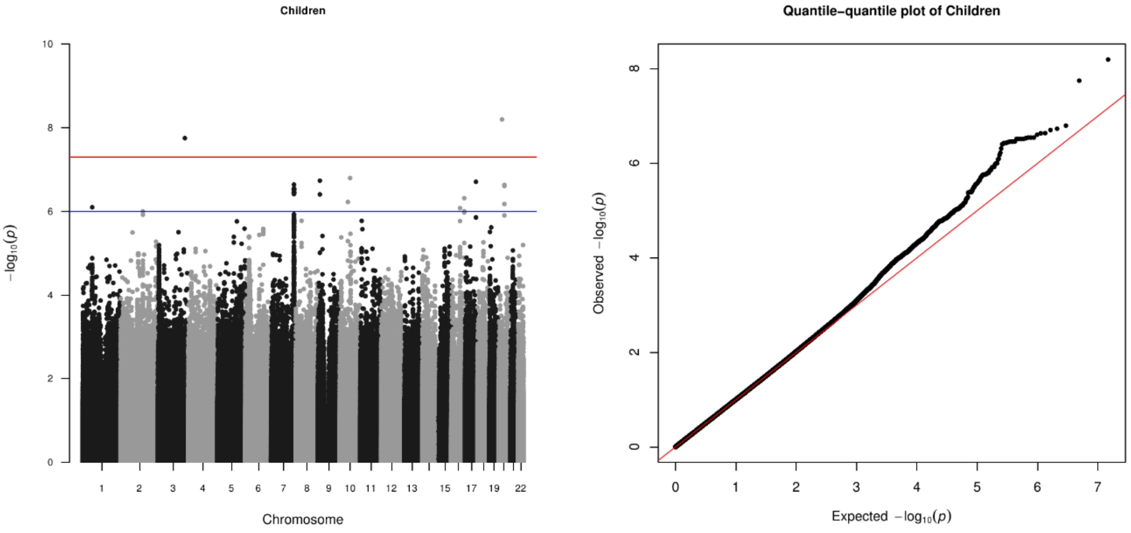

Figure S7. Manhattan (left) / QQ plots (right) for ALSPAC 6-9 year olds (□ =0.997).

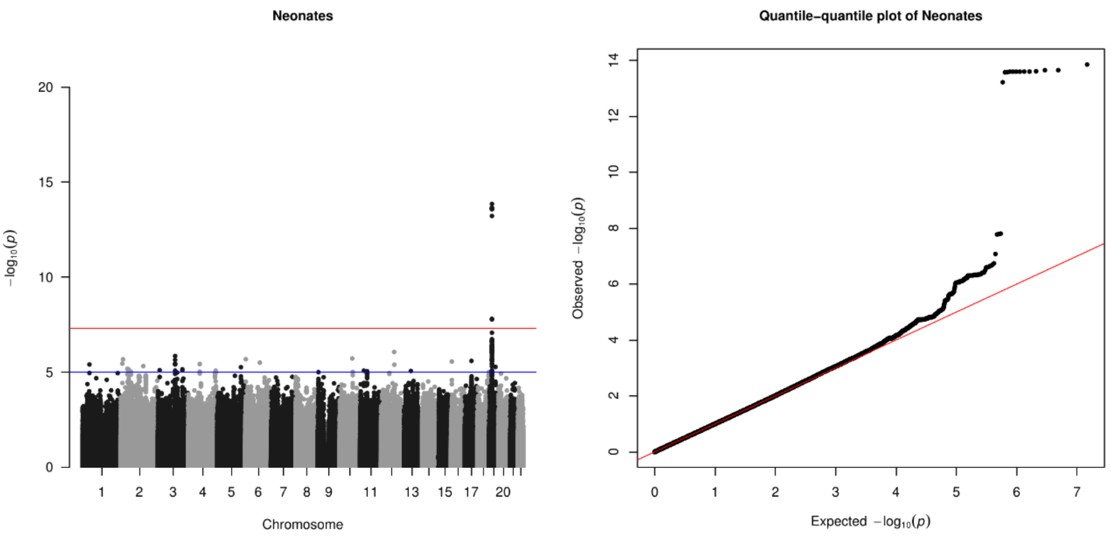

Figure S8. Manhattan (left) / QQ plots (right) for ALSPAC Neonates (□ =1.004).

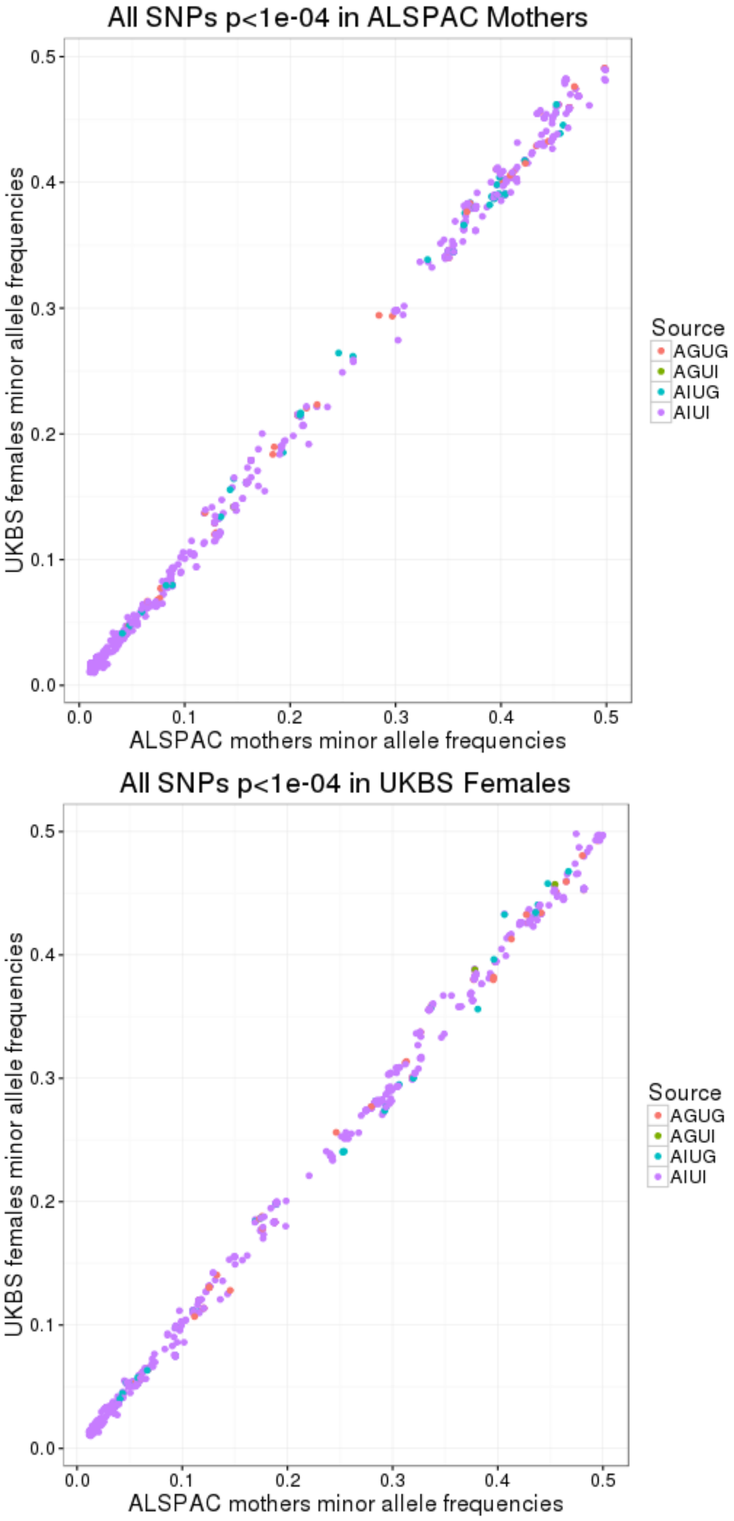

Figure S9. Correlations of minor allele frequencies between ALSPAC Mothers (n=5461) and UKBS Females (n=1338) for all hits p<1e-04, separately by cohort (top=ALSPAC Mothers, bottom=UKBS Females). AGUG=SNP genotyped in ALSPAC, genotyped in UKBS; AGUI= SNP genotyped in ALSPAC, imputed in UKBS; AIUG= SNP imputed in ALSPAC, genotyped in UKBS; AIUI= SNP imputed in ALSPAC, imputed in UKBS.
